# A Simple and Efficient Mechanochemical Route for the Synthesis of Salophen Ligands and of the Corresponding Zn, Ni, and Pd Complexes

**DOI:** 10.3390/molecules24122314

**Published:** 2019-06-22

**Authors:** Luca Leoni, Andrea Carletta, Luca Fusaro, Jean Dubois, Nikolay A. Tumanov, Carmela Aprile, Johan Wouters, Antonella Dalla Cort

**Affiliations:** 1 Università di Roma La Sapienza, Piazzale Aldo Moro 5, 00185 Roma, Italy; luca.leoni@uniroma1.it; 2 Namur Institute of Structured Matter (NISM) and Namur Research Institute for Life Sciences (NARILIS), University of Namur, 61 rue de Bruxelles, B-5000 Namur, Belgium; andrea.carletta@unamur.be (A.C.); luca.fusaro@unamur.be (L.F.); jean.dubois@unamur.be (J.D.); nikolay.tumanov@unamur.be (N.A.T.); carmela.aprile@unamur.be (C.A.)

**Keywords:** salophen ligands, metal salophen complexes, mechanochemistry

## Abstract

A number of salophen ligands and their Zn, Ni, and Pd complexes were synthesized by an efficient one-pot mechanosynthesis protocol. The reaction products were characterized by means of complementary solid-state techniques, i.e., powder X-ray diffraction, single-crystal X-ray diffraction, and solid-state NMR spectroscopy. Four new crystal structures of metal salophen complexes as DMSO solvates are here reported. The described simple and relatively fast (about 1 h for all derivatives) procedure is a good alternative to classical methods performed in organic solvents.

## 1. Introduction

The synthesis and study of transition metal complexes with organic ligands is an active area of research. One of the major reasons for such interest is that these compounds possess unique properties that lead to several applications widely reported in recent literature [1,2,3,4]. Hence, the easy, straightforward, sustainable, and efficient synthesis of such complexes is indeed highly desirable. A way to approach the problem can be the use of mechanosynthesis, i.e., the use of mechanical energy to trigger chemical reactions between solids [5,6]. This kind of protocol can be a valid tool for ligand preparation, as well as for the synthesis of the corresponding metal complexes [7,8]. The obvious advantages of using such an approach are the relatively large quantity of starting materials that can be used, the shorter reaction times, and the reduction of side reactions [6,9]. Mixer mills are the classical equipment used for this type of synthesis [10,11]. The absence of solvent, or its use in a limited amount (liquid-assisted grinding, LAG, technique), makes mechanosynthesis a greener alternative to conventional synthetic methods. Recently, this strategy was extensively used to produce cocrystals [12,13,14], solid solutions, and polymorphs [15,16,17], as well as metal–organic frameworks [18].

In the frame of our research project focused on the study of metal salophen and salen complexes as supramolecular receptors [19,20,21,22,23], we apply the mechanochemistry approach to the synthesis of such derivatives.

Salophen ((*N*,*N*-phenylene-bis(salicylimine)) and salen (*N*,*N*-bis(salicylidene) ethylenediamine) ligands are broadly used in coordination chemistry [24]. They are tetradentate Schiff bases derived from the condensation in organic solvents of 1,2-phenylenediamine or ethylenediamine with two equivalents of a salicylaldehyde derivative. The synthesis, when carried out in the presence of metal salts, leads directly to the isolation of the corresponding metal complex.

**Figure 1:**
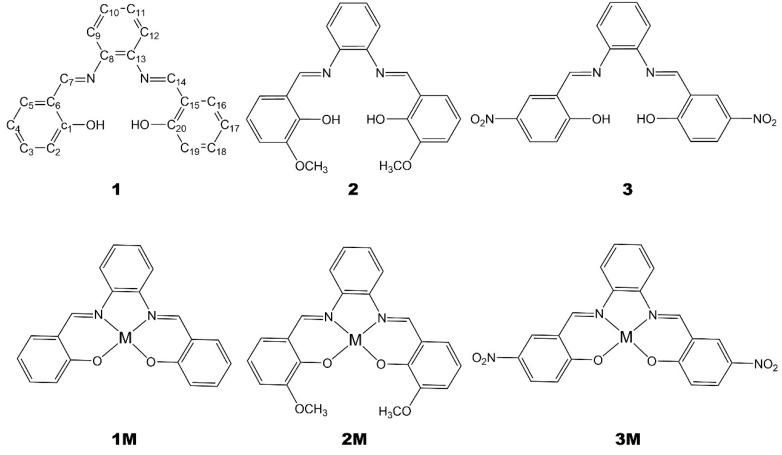
Ligands and corresponding metal complexes under study. Numbering scheme is provided for derivative **1** (**M** = **Zn, Ni, Pd**)**.**

Herein we report the alternative one-pot mechanochemical synthesis of a number of salophen ligands, **1**–**3**, and of the corresponding metal (M = Zn^2+^, Ni^2+^, Pd^2+^) complexes, **1M**–**3M**, Figure 1, representing a convenient, rapid, and sustainable approach to the synthesis of such derivatives.

## 2. Results

### 2.1. Synthesis

**Figure 2:**
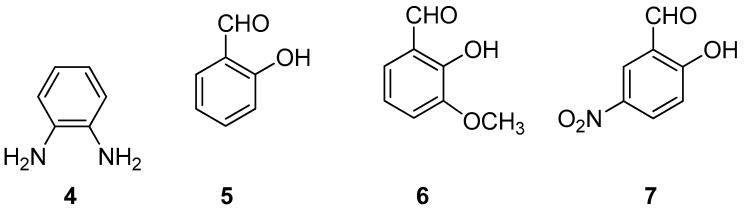
Structures of the building blocks used in the synthesis of ligands **1**–**3**.

For the synthesis of ligands, **1**–**3**, we started from *o*-phenylenediamine, **4**, and salicylaldehyde, **5**, *o*-vanillin, **6**, or 2-hydroxy-5-nitrobenzaldehyde, **7**, Figure 2. The solid mixture was ground to obtain the corresponding ligand. To obtain metal–salophen complexes, **1M**–**3M,** we added to the previous mixture, from the beginning, Zinc(II) or Nickel(II) acetate, (Zn(OAc)_2_, Ni(OAc)_2_), or Palladium(II)-2,4-pentanedionate, C_10_H_16_O_4_Pd. We report here procedures and characterizations of the isolated products.

**Figure 3:**
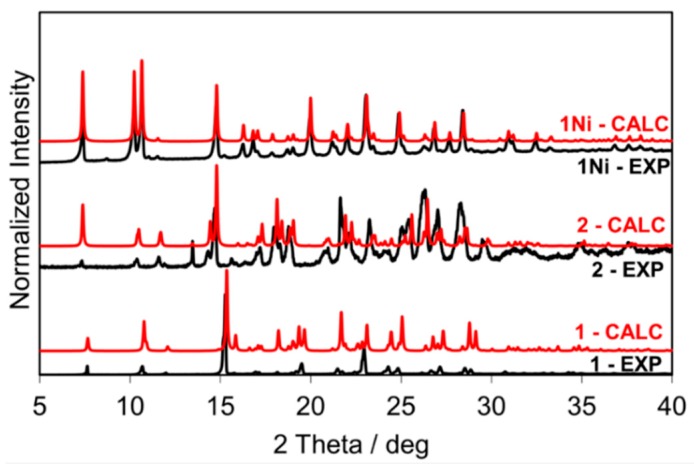
From bottom to top: PXRD patterns of **1**, **2**, and **1Ni** (calculated powder patterns in red, experimental powder patterns in black).

Salophen ligand **1** (yield 70%) was prepared by kneading 1 mmol of *o*-phenylenediamine, **4**, and 2 mmol of salicylaldehyde, **5**, obtaining a yellow powder. Its ^1^H-NMR confirmed the formation of the pure product (Figure S1). The crystal structure of the salophen ligand **1** was already reported by Reyes-Gutiérrez et al. [25], Cambridge Structure Database (CSD) ref code: EKEYEA, Figure 3. The experimental powder X-ray diffraction (PXRD) pattern of the isolated product matched the simulated powder pattern of EKEYEA, Figure 3.

**Figure 4:**
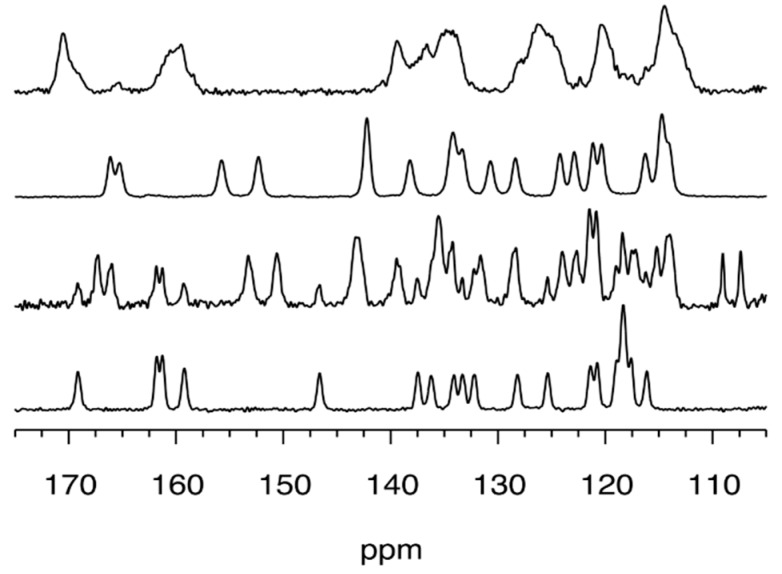
**^13^C** solid-state NMR spectra (105–175 ppm region) recorded at 11.7 T, and room temperature of mechanosynthesized ligand **1** and of the corresponding metal–salophen complexes (from bottom to top: compound **1**, **1Pd**, **1Ni**, **1Zn**).

Definitive evidence about the formation of this compound was obtained by solid-state NMR (SSNMR) spectroscopy. The ^13^C cross-polarization (CP-MAS) spectrum showed sharp signals of **1,** Figure 4, and no signals belonging to starting compounds.

Compound **2** (yield 60%) was synthesized by LAG (four drops of MeOH were added per gram of mixture) from 1 mmol of *o*-phenylenediamine, **4**, and 2 mmol of *o*-vanillin, **6**. The liquid-state ^1^H-NMR of the obtained orange powder is reported in Figure S2. Comparison of the X-ray powder diffraction pattern of **2** with the one simulated from its single-crystal structure (CSD entry MEPWUA [26]) revealed near-total conversion of reactants into compound **2** phase.

Attempts to mechanosynthesize ligand **3**, from 1 mmol of o-phenylenediamine, **4**, and 2 mmol of 2-hydroxy-5-nitrobenzaldehyde, **7**, were unsuccessful by both dry and liquid-assisted grinding and led to a mixture of the starting compounds.

In our hands, the successful outcome of metal–salophen complex syntheses seemed to be strictly related to the nature of metal counteranion. Best results were obtained using acetate for Zn and Ni, and 2,4-pentanedionate for Pd. The use of chloride, i.e., a weaker base, led to the recovery of a mixture of starting materials and ligands in all cases.

Compound **1Zn** was prepared (yield 62%) by kneading 1 mmol of *o*-phenylenediamine, **4**, 2 mmol of salicylaldehyde, **5**, and 1.2 mmol of Zn(OAc)_2_. The ^1^H-NMR, Figure S3, revealed a spectrum consistent with the expected compound. Comparison with data from the literature of the powder pattern of **1Zn** was not possible, since its single-crystal structure is not reported in the CSD, nor was determination from powder achievable, given the low crystallinity of the compound. Furthermore, several attempts to obtain crystals suitable for X-ray analysis were unsuccessful. The SSNMR spectra, Figure 4, showed the absence of resonances belonging to the free salophen ligand **1**. The peaks are broader than those of the corresponding ligand, as expected for a compound characterized by a low degree of crystallinity.

Compound **1Ni** was synthesized by kneading 1 mmol of *o*-phenylenediamine, **4**, 2 mmol of salicylaldehyde, **5**, and 1.2 mmol of Ni(OAc)_2_ (yield 68%). The product had a red clay-like color. ^1^H-NMR, Figure S4, confirmed the formation of the compound. Experimental powder pattern matches that were calculated from single-crystal data (CSD ref code: ZZZTZI02 [27], see Figure 3), and SSNMR, Figure 4, supported the assignment.

**Figure 5:**
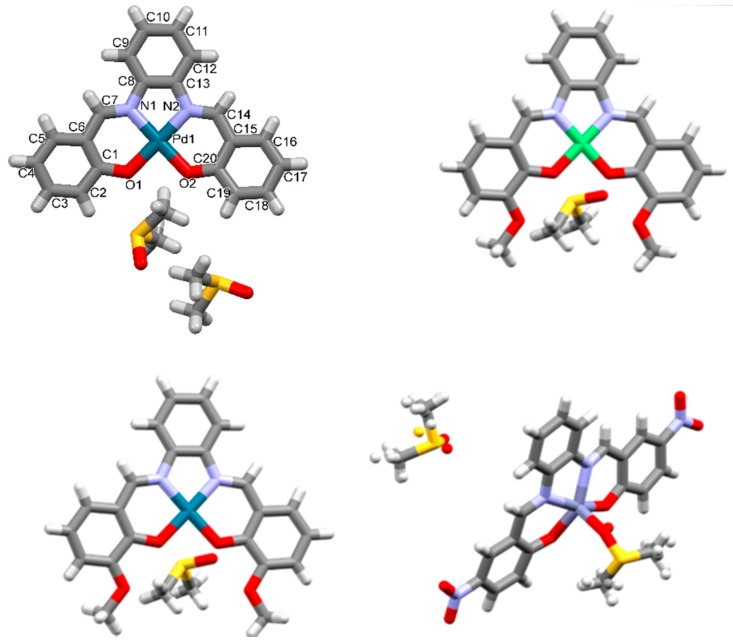
Asymmetric unit of **1Pd**, top left, **2Ni**, top right, **2Pd** bottom left, and **3Zn**, bottom right, obtained as DMSO solvates and adducts (shown in capped stick representation). Crystallographic labelling scheme is provided for **1Pd.**

Compound **1Pd** was prepared by kneading 1 mmol of *o*-phenylenediamine, **4**, 2 mmol of salicylaldehyde, **5**, and 1.2 mmol of Pd(II) 2,4-pentanedionate. SSNMR spectrum, Figure 4, showed that the reaction was uncomplete since both peaks of ligand **1** and product **1Pd** were detected. Peaks and chemical shift values observed in the SSNMR spectrum of **1Pd** were similar to those registered for **1Ni,** suggesting that the **1Pd** phase, in the recovered grinding mixture, belonged to the orthorhombic Pd-salophen complex, whose structure is reported in the CSD as PYSALP. Indeed PYSALP [28], **1Pd**, and ZZZTZI02, **1Ni,** were found to be isostructural, based on their cell parameters. The purification of **1Pd** by washing the crude with small amounts of methanol and ethanol was not successful. Nevertheless, **1Pd** was obtained in a pure form as a DMSO solvate, ^1^H-NMR, Figure S5, by recrystallization from DMSO that led to the isolation of orange crystals suitable for X-ray structure determination (crystal structure provided in Figure 5).

Compounds **2M**, M = Zn and Ni, were generated by dry grinding 1 mmol of *o*-phenylenediamine, **4**, 2 mmol of *o*-vanillin, **6**, and 1.2 mmol of the corresponding metal acetate. In the case of **2Pd**, LAG protocol was applied.

Compound **2Zn** (yield 64%), ^1^H-NMR is reported in Figure S6. Its SSNMR spectrum shows the disappearance of ligand peaks (Figure S10). Unfortunately, no suitable crystals for single-crystal X-ray diffraction (SCXRD) were obtained.

Compound **2Ni** (yield 65%), ^1^H-NMR is reported in Figure S7. Analysis by PXRD showed that compound **2Ni** is crystalline. Single crystals of the DMSO solvate suitable for structure determination by SCXRD were obtained from DMSO solution, see the structural section (crystal structure provided in Figure 5).

Complex **2Pd** was synthesized by LAG from 1 mmol of *o*-phenylenediamine, 2 mmol of *o*-vanillin, **6**, and 1.2 mmol of Palladium(II) 2,4-pentanedionate. Analogously to what was observed for compound **1Pd**, conversion was not complete. The longer reaction time did not lead to any improvement. The solid phase contained the **2Pd** tetrahydrate compound (CSD ref code: AVAVUP) [29]. Pure crystalline **2Pd** (DMSO solvate) was obtained by recrystallization in DMSO, as described in the structural section. The solution state ^1^H-NMR and ^13^C-NMR spectra of **2Pd** are provided in Figure S8.

Compound **3Zn** was synthesized by liquid-assisted grinding by adding four drops of MeOH to 1 mmol of *o*-phenylenediamine, 2 mmol of 2-hydroxy-5-nitrobenzaldehyde, and 1.2 mmol of Zn(OAc)_2_ (yield 85%, ^1^H-NMR, Figure S9). PXRD studies showed low crystallinity for this derivative. Yellow needle-like single crystals suitable for SCXRD were obtained by slow evaporation from a saturated solution in DMSO, leading to a DMSO solvate (crystal structure reported in Figure 5). SSNMR spectrum is reported in Figure S11.

### 2.2. Crystal Structure Details

All as-synthesized powder products were crystallized from DMSO saturated solutions by slow evaporation technique. All obtained solids are DMSO solvates.

#### 2.2.1. Solid **1Pd^.^(DMSO)_2_**

**Figure 6:**
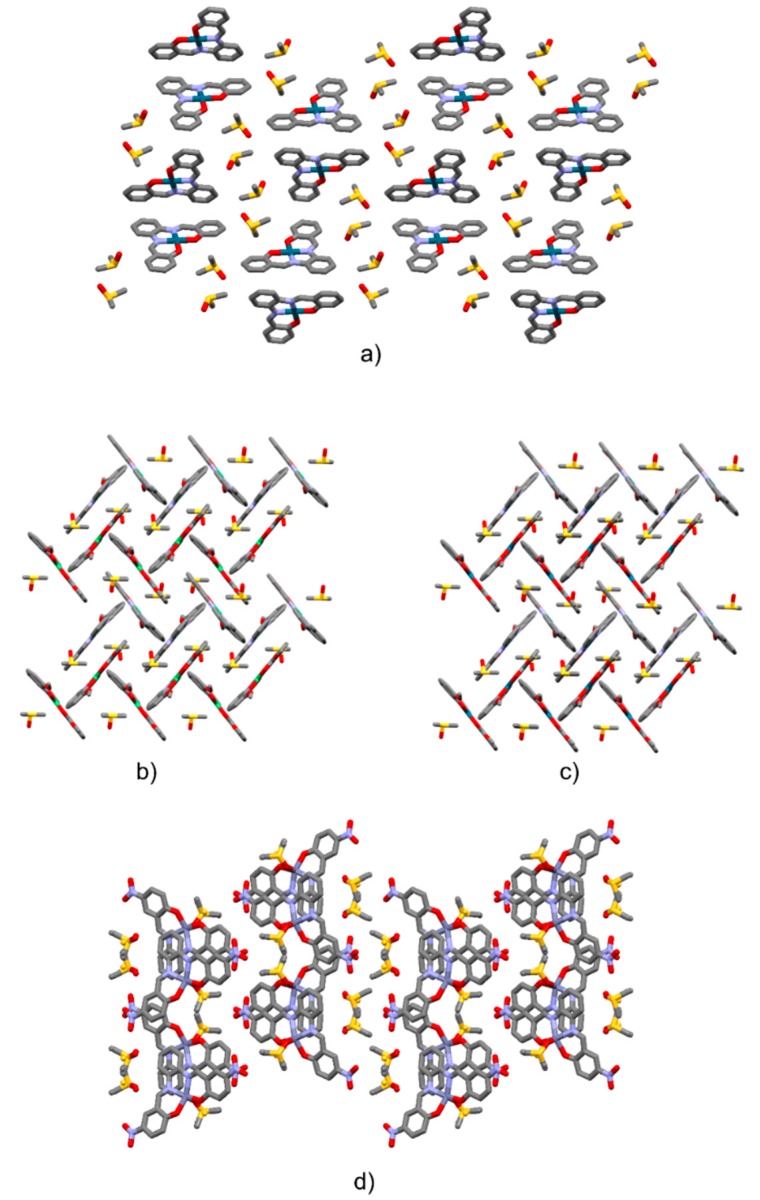
Crystal packing view of **1Pd^.^**(**DMSO**)**_2_** (**a**, view along the crystallographic *b*-axis); **2Ni^.^DMSO** (**b**, view along the [101] direction); **2Pd^.^DMSO** (**c**, view along the [101] direction); **3Zn(DMSO**)**_2_** (**d**, view along the *c*-axis). Hydrogen atoms are omitted for clarity.

Orange plates were obtained from a saturated DMSO solution after four days. A DMSO solvate was obtained, belonging to the *P*2_1_/*n* space group, with a 1:2 stoichiometry (Figure 5). In the crystal, **2Pd** molecules were stacked in a head-to-head parallel-displaced fashion along the crystallographic *b*-axis (Figure 6a and Figure S12). One of the DMSO molecules interacted through CH…O weak hydrogen bonds with the salophen complex (CH_DMSO_…O1 bond distance: 3.298(3) Å, C-H_DMSO_-O1 angle 132.8°; CH_DMSO_…O2 bond distance: 3.373(3) Å, C-H_DMSO_-O2 angle 139.3°).

#### 2.2.2. Solid **2Ni^.^DMSO**

Dark red plates were obtained from DMSO after four days. The crystals belonged to the monoclinic *P*2_1_/*c* space group. One molecule of **2Ni** was present in the asymmetric unit along with one molecule of DMSO. Molecules had a planar conformation (selected structural parameters are provided in Table S2). They were assembled in centrosymmetric dimers, which were staked in parallel-displaced fashion over a half of the molecule (parallel displacement and interplanar separation are provided in Figure 6b and Figure S13). In this way, the remaining half molecule was available for edge-to-face interactions with a second centrosymmetric dimer. The resulting packing assumed, then, a herringbone-like structure (see Figure 6b).

#### 2.2.3. Solid **2Pd^.^DMSO**

Red blocks were obtained from DMSO after four days. The crystals belonged to the monoclinic *P*2_1_/*c* space group. One molecule of **2Pd** is present in the asymmetric unit along with one molecule of DMSO. The molecule had a planar conformation. **2Pd^.^DMSO** was isostructural with **2Ni^.^DMSO** (see Figure 6c and Figure S13, and Table S1).

#### 2.2.4. Solid **3Zn^.^**(**DMSO**)**_2_**

Yellow plates were obtained from DMSO after four days. The crystals belonged to the monoclinic *P*2_1_/*c* space group. One molecule of **3Zn** was present in the asymmetric unit along with two disordered molecules of DMSO. One molecule of DMSO interacted with **3Zn** by non-covalent interactions. The second molecule of DMSO was, instead, bonded to the Zinc ion via its oxygen atom at 2.150(9) Å. Therefore, the metal center acted as a coordinating site. The geometry of the so-formed structure (with respect to the central Zn atom) was that of a distorted square pyramid (Table S3 for bond distances and angles). This behaviour of Zn–salophens is well known and various examples of water, pyridine, acetate, and DMF adducts are reported in the CSD [30,31,32,33,34]. Along the crystallographic *b*-axis **3Zn** molecules were assembled through glide plane operations. These so-formed columns formed layers along the crystallographic *c*-axis. Layers developed parallel to the *a*-axis and were related by two-fold screw axis symmetry operations (Figure 6d and Figure S14).

## 3. Materials and Methods

### 3.1. Reactants 

All reactants were purchased from Sigma–Aldrich and used as received. Solvents used for the washing step and crystallization (EtOH, MeOH, DMSO) are commercially available and were used without further purification.

### 3.2. Mechanosynthesis 

All compounds were synthesized by mechanosynthesis. Dry- and liquid-assisted grinding (LAG, in methanol) were performed by means of a Retsch MM 400 Mixer Mill in 2 mL Eppendorf tubes (8–10 stainless-steel grinding balls of 1 mm diameter for each sample). The operating frequency was set at 30 Hz and reactants were milled for 60 min. After milling, a washing step for the compounds was required. The as-synthesized products were washed with 3 mL of MeOH and 1 mL of EtOH.

#### 3.2.1. Powder X-Ray Diffraction 

X-ray powder patterns were collected in the 2θ range 5°–40° using a Panalytical X’Pert PRO diffractometer (Bragg-Brentano geometry, Cu Kα radiation, X’Celerator linear detector, step size 0.017°; 45 mA, 30 kV). The program Mercury was used for the calculation of X-ray powder patterns from single-crystal data.

#### 3.2.2. Single-Crystal X-Ray Diffraction (SCXRD)

Single-crystal X-ray diffraction data were collected at 100 K for **1Pd**^.^**(DMSO)_2_**, **2Ni**^.^**DMSO**, and **2Pd**^.^**DMSO**, and at 295 K for **3Zn**^.^**(DMSO)_2_** on an Oxford Diffraction Gemini Ultra R system (4-circle kappa platform, Ruby CCD detector) using Mo Kα (λ = 0.71073 Å) radiation for **1Pd^.^(DMSO)_2_**, **2Ni^.^DMSO**, and **2Pd^.^DMSO**, and Cu Kα (λ = 1.54184 Å) for **3Zn^.^(DMSO)_2_**. The structures were solved by SHELXT [35] and then refined by full-matrix least square refinement of |F|^2^ using SHELXL-2016 [36]. Non-hydrogen atoms were refined anisotropically. Hydrogen atoms were located from a difference Fourier map. Hydrogen atoms were refined in the riding mode with isotropic temperature factors fixed at 1.2Ueq of the parent atoms (1.5Ueq for the methyl group).

#### 3.2.3. Liquid-State NMR

Liquid NMR spectra were collected at 25 °C on a JEOL ECA spectrometer operating at 9.4 T (400 MHz) using DMSO-*d_6_* as solvent. The ^1^H chemical shift scale was calibrated using the residual signal of DMSO (2.50 ppm).

**Compound 1.** (yield = 70%) ^1^H-NMR δH (400 MHz, DMSO-*d_6_*), 12.93 (2H, s, OH), 8.91 (2H, s, CH), 7.64 (2H, d, CH, *J* = 8Hz), 7.45–7.36 (6 H, m, CH), 6.96–6.92 (4 H, m, CH). ^13^C-NMR δC (100 MHz, DMSO-*d_6_*), 164.1, 160.4, 142.3, 133.5, 132.5, 127.8, 119.8, 119.5, 119.1, 116.7.

**Compound 2.** (yield = 60%) ^1^H-NMR δH (400 MHz, DMSO-*d_6_*), 12.99 (2H, s, OH), 8.89 (2H, s, CH), 7.45–7.36 (4 H, m, CH), 7.22 (2H, d, CH, *J* = 8 Hz), 7.10 (2H, d, CH, *J* = 8 Hz), 6.88 (2H, t, CH, *J* = 8 Hz), 3.78 (6 H, s, OCH_3_). ^13^C-NMR δC (100 MHz, DMSO-*d_6_*), 164.9, 151.1, 148.4, 142.6, 128.3, 124.3, 120.3, 119.9, 119.1, 116.0, 56.2.

**Compound 1Zn.** (yield = 62%). ^1^H-NMR δH (400 MHz, DMSO-*d_6_*), 8.99 (2H, s, CH), 7.88–7.86 (2H, m, CH), 7.40–7.35 (4 H, m, CH), 7.21 (2H, t, CH, *J* = 8 Hz), 6.68 (2H, d, CH, *J* = 8 Hz), 6.48 (2H, t, CH, *J* = 8 Hz). ^13^C-NMR δC (100 MHz, DMSO-*d_6_*) 172.3, 162.9, 139.4, 136.2, 134.3, 127.3, 123.1, 119.4, 116.5, 112.9.

**Compound 1Ni.** (yield = 68%) ^1^H-NMR δH (400 MHz, DMSO-*d_6_*), 8.86 (2H, s, CH), 8.13–8.11 (2H, m, CH), 7.57 (2H, d, CH, *J* = 8 Hz), 7.32–7.27 (4 H, m, CH), 6.85 (2H, d, CH, *J* = 8 Hz), 6.64 (2H, t, CH, *J* = 8 Hz). ^13^C-NMR δC (100 MHz, DMSO-*d_6_*) 165.8, 157.1, 142.9, 135.7, 134.8, 128.2, 120.8, 120.7, 116.7, 115.8.

**Compound 1Pd.**^1^H-NMR δH (400 MHz, DMSO-*d_6_*), 9.17 (2H, s, CH), 8.34–8.30 (2H, m, CH), 7.71 (2H, d, CH, *J* = 8 Hz), 7.43–7.42 (4 H, m, CH), 6.99 (2H, d, CH, *J* = 8 Hz), 6.69 (2H, t, CH, *J* = 8 Hz). ^13^C-NMR δC (100 MHz, DMSO-*d_6_*) 166.6, 155.5, 143.7, 136.8, 136.7, 128.7, 121.4, 121.2, 117.7, 115.8.

**Compound 2Zn.** (yield = 64%) ^1^H-NMR δH (400 MHz, DMSO-*d_6_*), 8.98 (2H, s, CH), 7.87–7.85 (2H, m, CH), 7.35–7.33 (2H, m, CH), 6.99 (2H, d, CH, *J* = 8 Hz), 6.83 (2H, d, CH, *J* = 8 Hz), 6.40 (2H, t, CH, *J* = 8 Hz), 3.73(6 H, s, OCH_3_). ^13^C-NMR δC (100 MHz, DMSO-*d_6_*) 164.2, 163.3, 153.0, 139.9, 127.9, 127.6, 119.2, 116.9, 114.3, 112.3, 55.7.

**Compound 2Ni.** (yield = 65%) ^1^H-NMR δH (400 MHz, DMSO-*d_6_*), 8.87 (2H, s, CH), 8.12–8.10 (2H, m, CH), 7.30–7.29 (2H, m, CH), 7.17 (2H, d, CH, *J* = 8 Hz), 6.85 (2H, d, CH, *J* = 8 Hz), 6.54 (2H, t, CH, *J* = 8 Hz), 3.72 (6H, s, OCH_3_). ^13^C-NMR δC (100 MHz, DMSO-*d_6_*) 157.6, 156.9, 151.2, 142.8, 128.0, 125.9, 120.7, 116.7, 115.5, 115.1, 56.2.

**Compound 2Pd.**^1^H-NMR δH (400 MHz, DMSO-*d_6_*), 9.14 (2H, s, CH), 8.33–8.32 (2H, m, CH), 7.41–7.39 (2H, m, CH), 7.29 (2H, d, CH, *J* = 8 Hz), 6.98 (2H, d, CH, *J* = 8 Hz), 6.60 (2H, t, CH, *J* = 8 Hz), 3.78 (6H, s, OCH_3_). ^13^C-NMR δC (100 MHz, DMSO-*d_6_*) 157.8, 154.9, 150.9, 143.1, 128.0, 127.1, 120.5, 117.2, 114.9, 114.4, 55.3.

**Compound 3Zn.** (yield = 85%) ^1^H-NMR δH (400 MHz, DMSO-*d_6_*), 9.18 (2H, s, CH), 8.57 (2H, d, CH, *J* = 3 Hz), 8.05 (2H, dd, CH, *J_1_* = 8 Hz, *J_2_* = 3 Hz), 7.94 (2H, m, CH), 7.45 (2H, m, CH), 6.74 (2H, d, CH, *J* = 8 Hz). ^13^C-NMR δC (100 MHz, DMSO-*d_6_*) 177.2, 163.2, 139.5, 134.9, 134.6, 129.1, 129.0, 124.3, 119.1, 117.8.

#### 3.2.4. Solid-State NMR 

^13^C CP-MAS spectra were collected at room temperature on a Bruker Avance 500 spectrometer operating at 11.7 T (125 MHz for ^13^C) using a 4mm CP-MAS probe and a spinning frequency of 10 kHz. All the spectra were recorded using 512 averaged transients, a recycle delay of 5 s, and contact time of 2 ms. The chemical shift scale was referenced externally to solid adamantane (38.48 ppm and 29.45 ppm) [37] with respect to TMS.

## 4. Conclusions

The operational simplicity and short reaction times (of about 1 h for all derivatives) make this protocol quite useful, i.e., a handy method to obtain salophen ligands and corresponding metal complexes. The mechanochemical protocol here applied can be considered as a good alternative to classical methods that employ organic solvents. The obtained compounds here reported were fully characterized by NMR spectroscopy, PXRD, and SCXRD whenever possible. Four new crystal structures (DMSO solvates) were reported.

Furthermore, **3Zn**, synthesized through this procedure, is a quite interesting derivative that showed intriguing applications. It possesses biological activity due to the strong interaction with free plasmid DNA, showing very low cytotoxicity [38]. The easy synthetic availability through this protocol of series of such derivatives will allow further investigations on these aspects that seem to be related to the electronic properties of the substituents at the 5,5′ positions of the ligand.

X-ray crystallography and electronic data were deposited at the CCDC under deposition numbers 1844978-1844981.

## Supplementary Materials

The following are available online at https://www.mdpi.com/1420-3049/24/12/2314/s1, Figures S1–S9: solution ^1^H NMR (400 MHz, DMSO-*d_6_*) and ^13^C-NMR (100 MHz, DMSO-*d_6_*) spectra, Figures S10 and S11 solid state NMR data, Table S1: Crystallographic data for reported compounds, Table S2: Bond distances of the compounds under study, Table S3: Selected geometries for the distorted square pyramid observed in **3Zn****^.^****(DMSO)_2_** (M = Zn).
