# Supplementary material for: A Simple and Efficient Mechanochemical Route for the Synthesis of Salophen Ligands and of the Corresponding Zn, Ni, and Pd Complexes
Source: Molecules. 2019 Jun 22;24(12):2314. doi: 10.3390/molecules24122314 (PMC6631197; doi:10.3390/molecules24122314)
Supplement: Supplementary file 1 [file molecules-24-02314-s001.pdf]

Supplementary Materials

Article

A Simple and Efficient Mechanochemical Route for the Synthesis of Salophen Ligands and of the Corresponding Zn, Ni, and Pd Complexes

Luca Leoni [1], Andrea Carletta [2], Luca Fusaro [2], Jean Dubois [2], Nikolay A. Tumanov [2], Carmela Aprile [2], Johan Wouters [2,*] and Antonella Dalla Cort [1,*]

1 Università di Roma La Sapienza, Piazzale Aldo Moro 5, Roma 00185, Italy; luca.leoni@uniroma1.it
2 Namur Institute of Structured Matter (NISM) and Namur Research Institute for Life Sciences (NARILIS), University of Namur, 61 rue de Bruxelles, Namur B-5000, Belgium; andrea.carletta@unamur.be (A.C.); luca.fusaro@unamur.be (L.F.); jean.dubois@unamur.be (J.D.); nikolay.tumanov@unamur.be (N.A.T.); carmela.aprile@unamur.be (C.A.)
* Correspondence: johan.wouters@unamur.be (J.W.); antonella.dallacort@uniroma1.it (A.D.C.); Tel.: +32 81 724550 (J.W.); +39 06 49913087 (A.D.C.)

Compound 1

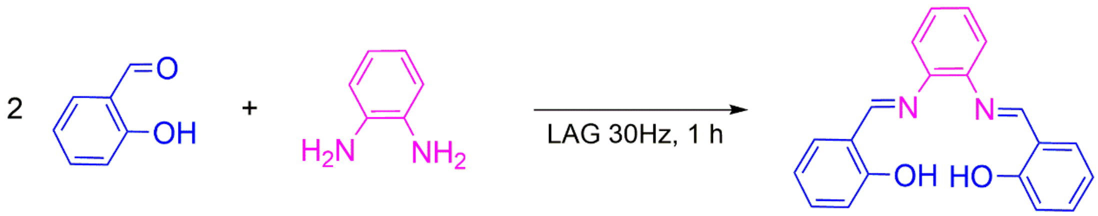

1

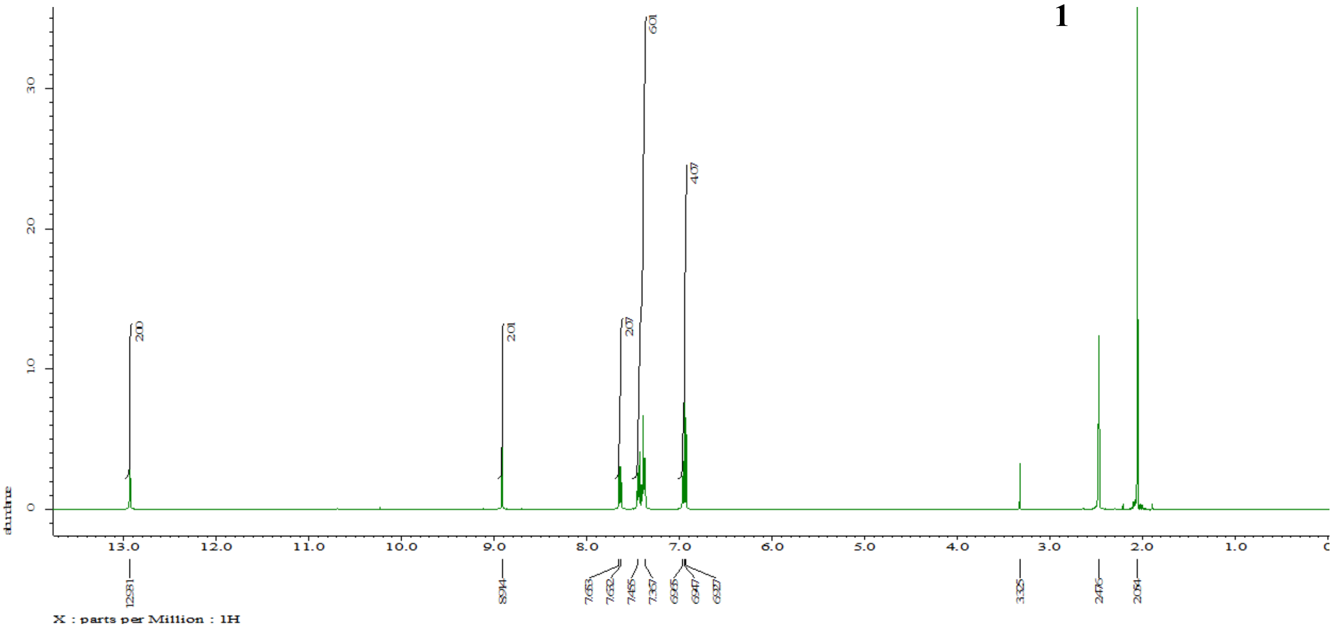

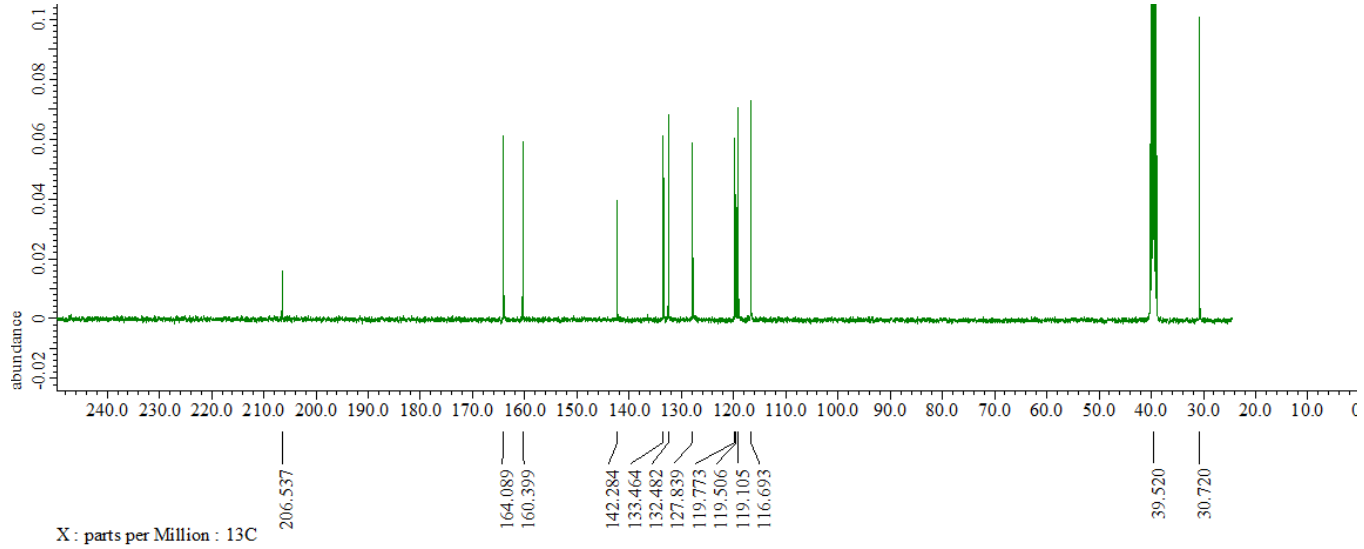

Figure 1. ^{1}H NMR (400 MHz, DMSO-d_6) and ^{13}C NMR (100 MHz, DMSO-d_6) spectra of compound **1.** Top: **^{1}H NMR** δH, 12.93 (2 H, s, OH), 8.91 (2H, s, CH), 7.64 (2 H, d, CH, J = 8Hz), 7.45-7.36 (6 H, m, CH), 6.96-6.92 (4 H, m, CH). Bottom: **^{13}C NMR** δC, 164.1, 160.4, 142.3, 133.5, 132.5, 127.8, 119.8, 119.5, 119.1, 116.7.

NMR spectra of this compound is in agreement with that reported in the literature. [1]

Compound 2

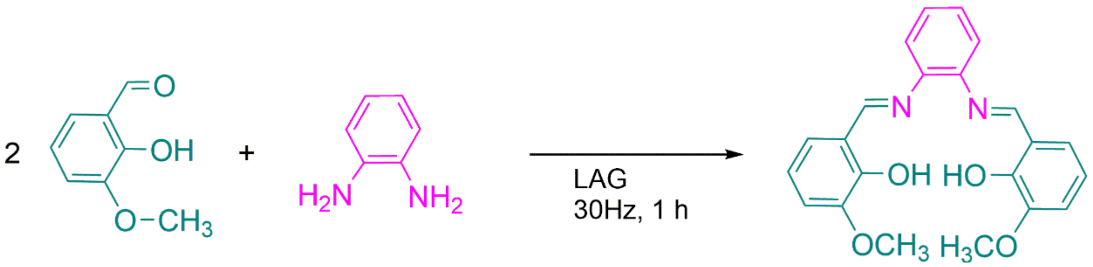

2

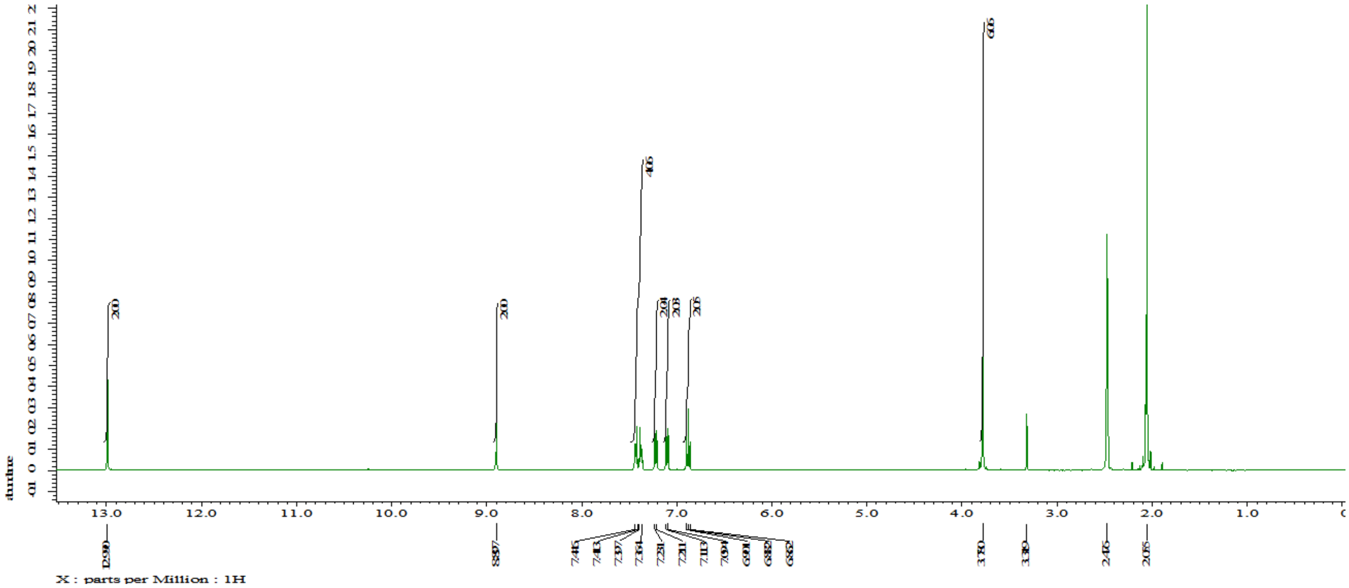

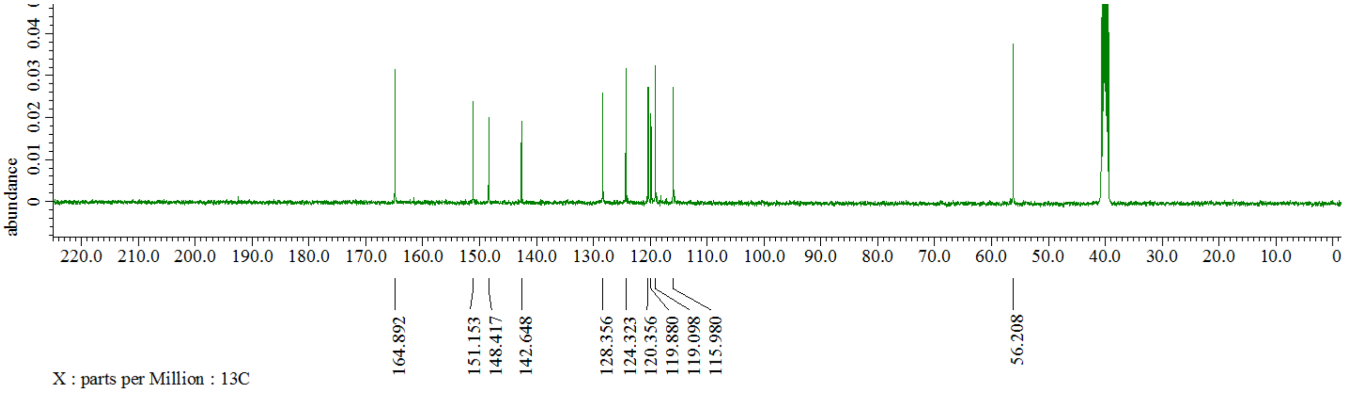

Figure S1 . ^{1}H NMR (400 MHz, DMSO-d_6) and **^{13}C-NMR** (100 MHz, DMSO-d_6) spectra of compound **2.** **^{1}H-NMR** δH (400 MHz, DMSO-d_6), 12.99 (2 H, s, OH), 8.89 (2H, s, CH), 7.45-7.36 (4 H, m, CH), 7.22 (2 H, d, CH, J = 8 Hz), 7.10 (2 H, d, CH, J = 8 Hz), 6.88 (2 H, t, CH, J = 8 Hz), 3.78 (6 H, s, OCH3). **^{13}C-NMR** δC (100 MHz, DMSO-d_6), 164.9, 151.1, 148.4, 142.6, 128.3, 124.3, 120.3, 119.9, 119.1, 116.0, 56.2).

NMR spectra of this compound is in agreement with that reported in the literature. [1]

Compound 1Zn

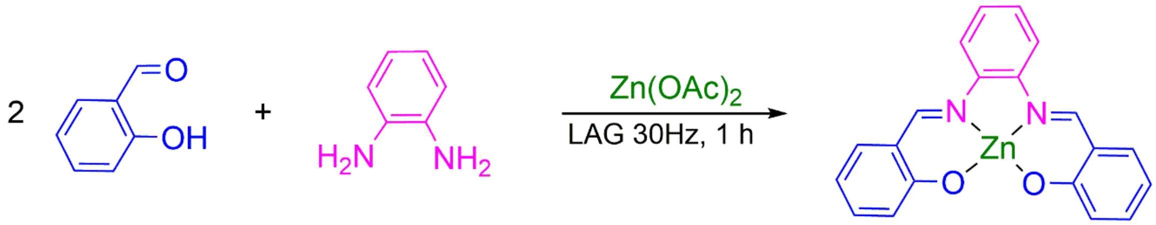

1Zn

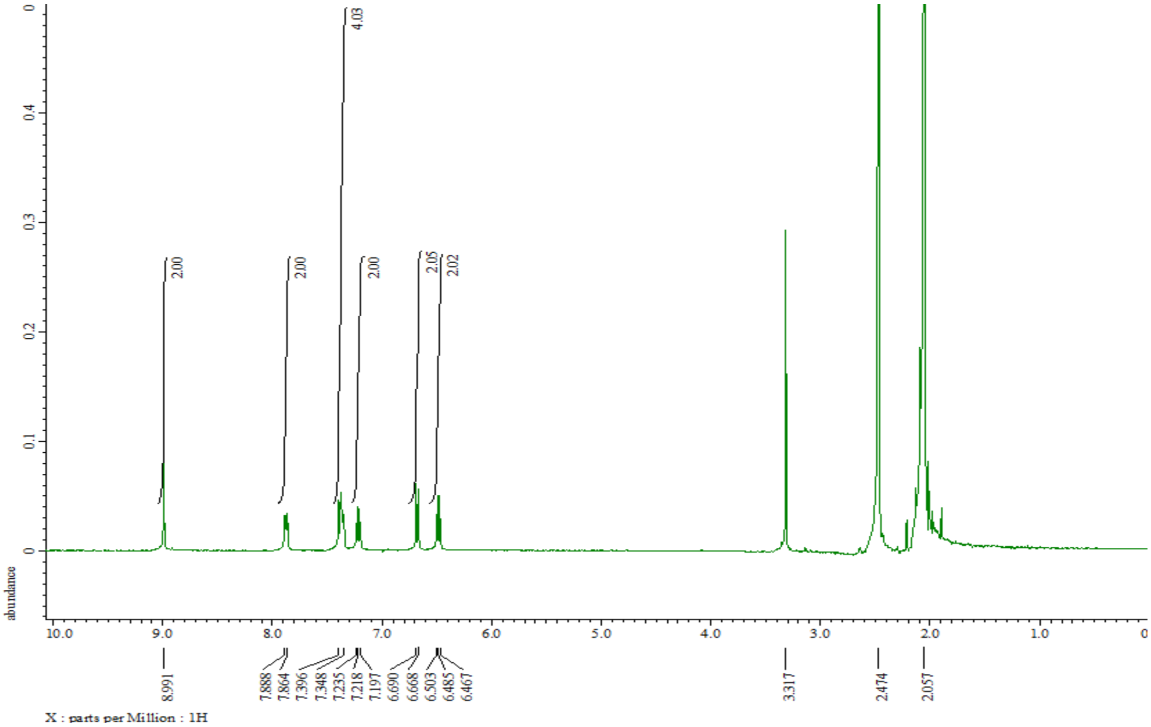

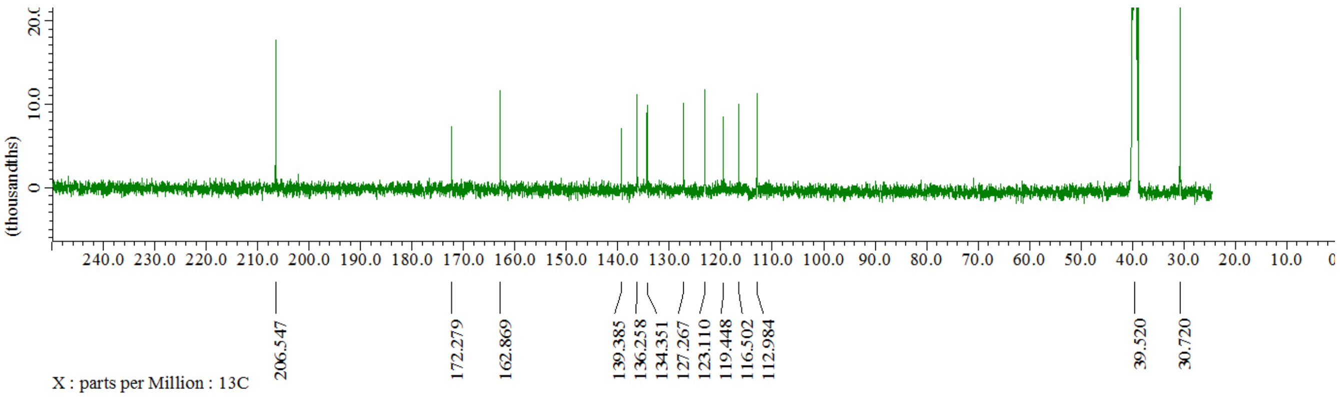

Figure S2. ^{1}H NMR (400 MHz, DMSO-d_6) and **^{13}C-NMR** (100 MHz, DMSO-d_6) spectra of compound **1Zn. ^{1}H NMR** δH (400 MHz, DMSO-d_6), 8.99 (2H, s, CH), 7.88-7.86 (2 H, m, CH), 7.40-7.35 (4 H, m, CH), 7.21 (2 H, t, CH, J = 8 Hz), 6.68 (2 H, d, CH, J = 8 Hz), 6.48 (2 H, t, CH, J = 8 Hz). **^{13}C NMR** δC (100 MHz, DMSO-d_6) 172.3, 162.9, 139.4, 136.2, 134.3, 127.3, 123.1, 119.4, 116.5, 112.9).

NMR spectra of this compound is in agreement with that reported in the literature. [2]

Compound 1Ni

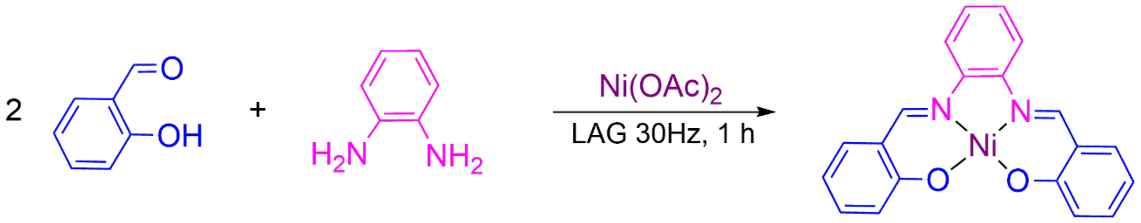

1Ni

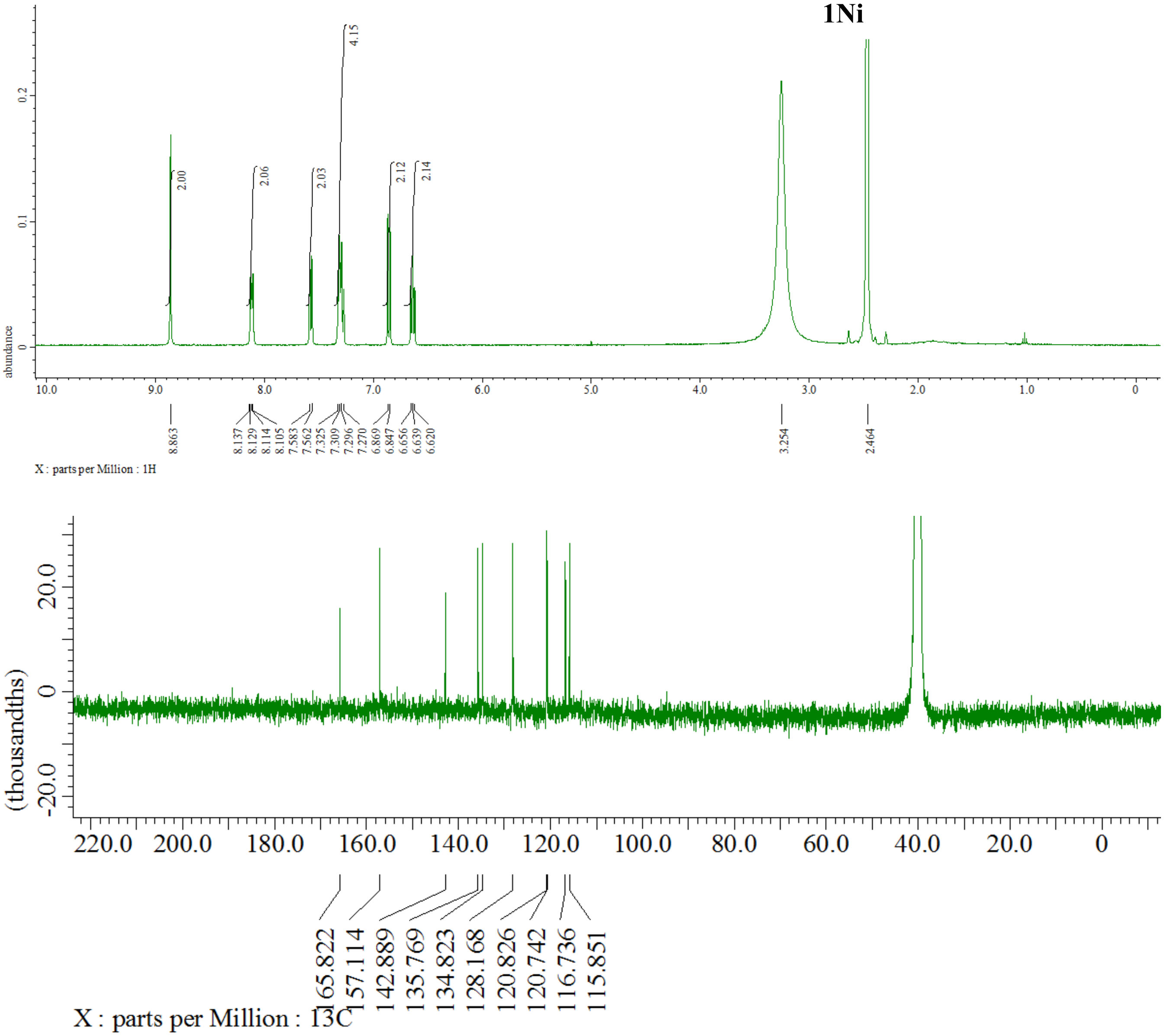

Figure S3 . ^{1}H NMR (400 MHz, DMSO-*d*$_6$) and ^{13}C-NMR (100 MHz, DMSO-*d*$_6$) spectra of compound **1Ni**. **^{1}H NMR** δH (400 MHz, DMSO-*d*$_6$), 8.86 (2H, s, CH), 8.13-8.11 (2 H, m, CH), 7.57 (2 H, d, CH, J = 8Hz), 7.32-7.27 (4 H, m, CH), 6.85 (2 H, d, CH, J = 8Hz), 6.64 (2 H, t, CH, J = 8 Hz). **^{13}C NMR δC** (100 MHz, DMSO-*d*$_6$) 165.8, 157.1, 142.9, 135.7, 134.8, 128.2, 120.8, 120.7, 116.7, 115.8).

Compound 1Pd

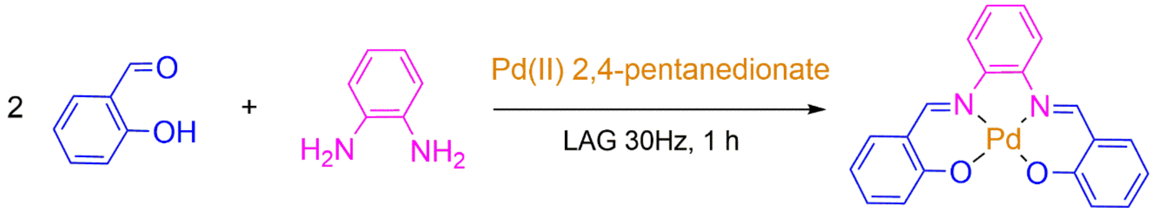

1Pd

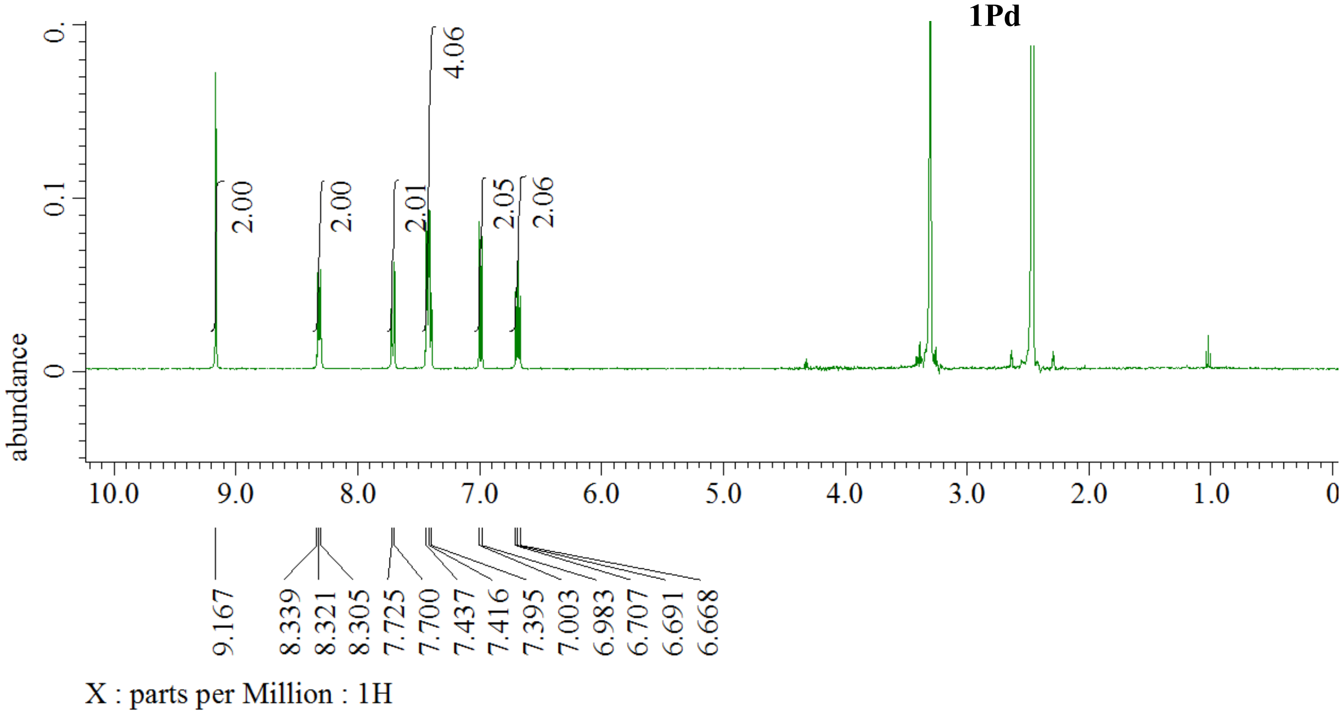

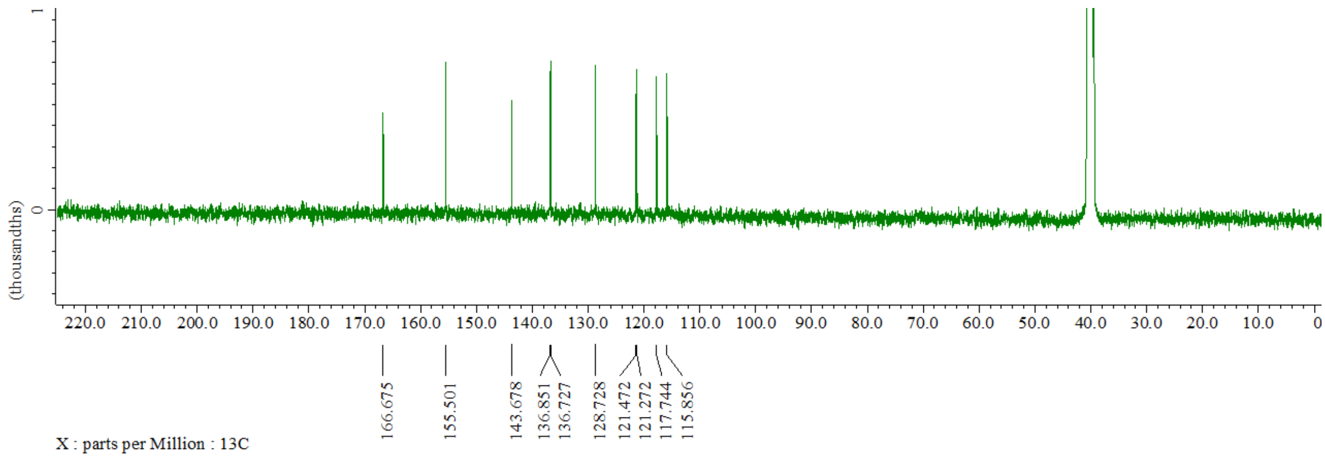

Figure S4. ^{1}H NMR (400 MHz, DMSO-d_6) and ^{13}C-NMR (100 MHz, DMSO-d_6) spectra of compound **1Pd**. ^{1}H NMR δH (400 MHz, DMSO-d_6), 9.17 (2H, s, CH), 8.34-8.30 (2 H, m, CH), 7.71 (2 H, d, CH, J = 8Hz), 7.43-7.42 (4 H, m, CH), 6.99 (2 H, d, CH, J = 8Hz), 6.69 (2 H, t, CH, J = 8 Hz). ^{13}C NMR δC (100 MHz, DMSO-d_6) 166.6, 155.5, 143.7, 136.8, 136.7, 128.7, 121.4, 121.2, 117.7, 115.8).

NMR spectra of this compound is in agreement with that reported in the literature. [3]

Compound 2Zn

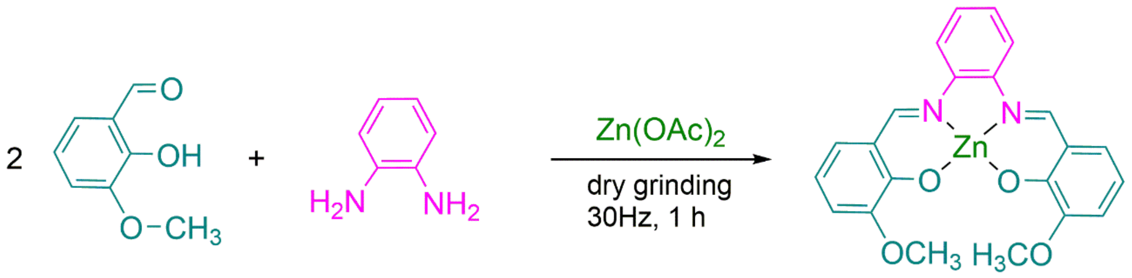

2Zn

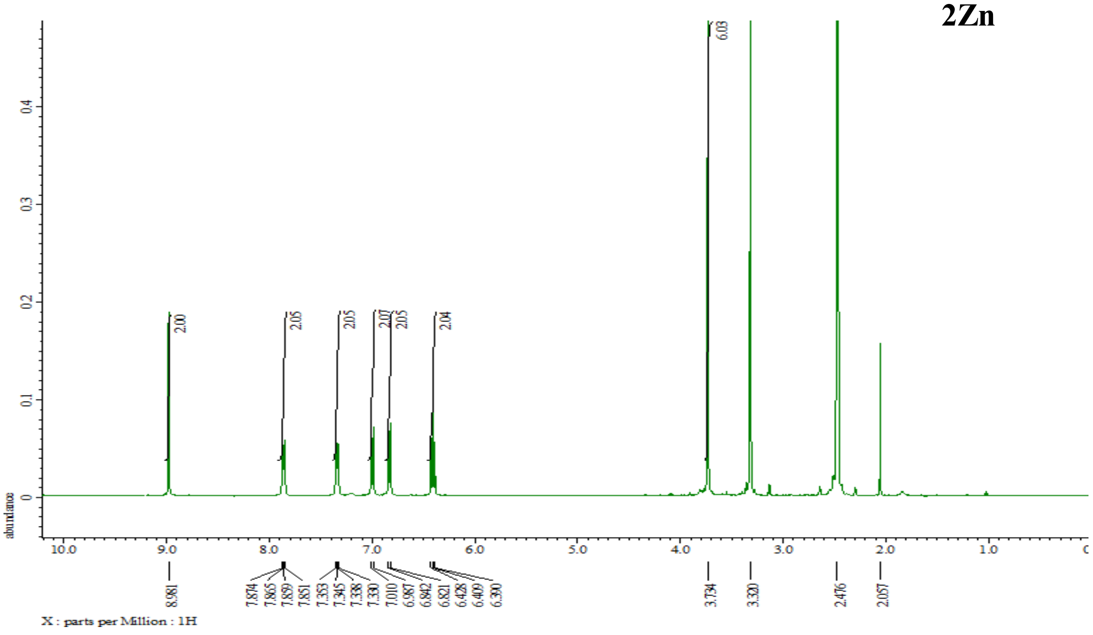

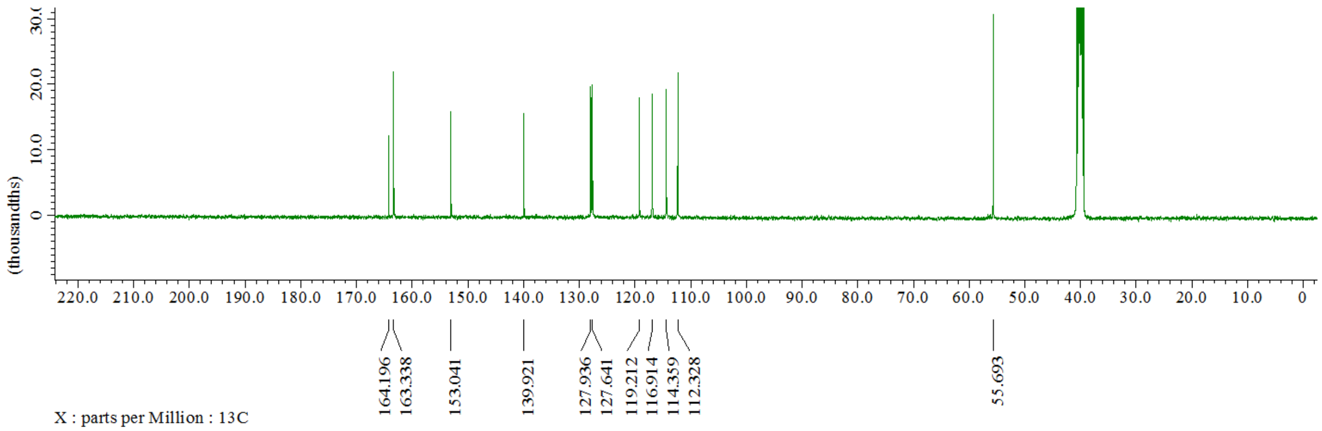

Figure S5. ^{1}H NMR (400 MHz, DMSO-d_6) and ^{13}C-NMR (100 MHz, DMSO-d_6) spectra of compound **2Zn**. **^{1}H NMR** δH (400 MHz, DMSO-d_6), 8.98 (2H, s, CH), 7.87-7.85 (2 H, m, CH), 7.35-7.33 (2 H, m, CH), 6.99 (2 H, d, CH, J = 8 Hz), 6.83 (2 H, d, CH, J = 8 Hz), 6.40 (2 H, t, CH, J = 8 Hz), 3.73(6 H, s,

OCH3). **^{13}C NMR** δC (100 MHz, DMSO-d_6) 164.2, 163.3, 153.0, 139.9, 127.9, 127.6, 119.2, 116.9, 114.3, 112.3, 55.7).

NMR spectra of this compound is in agreement with that reported in the literature. [4]

Compound 2Ni

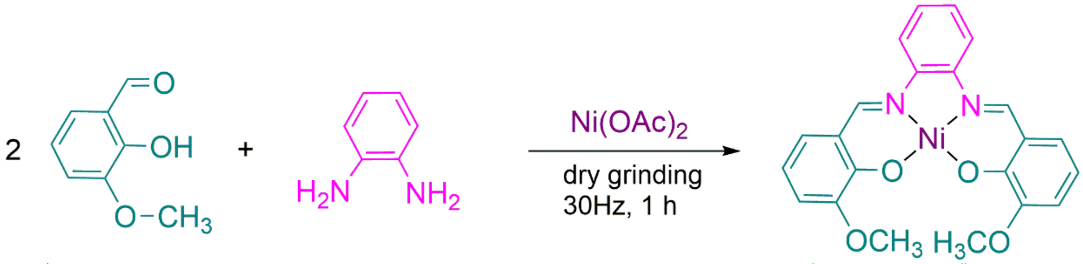

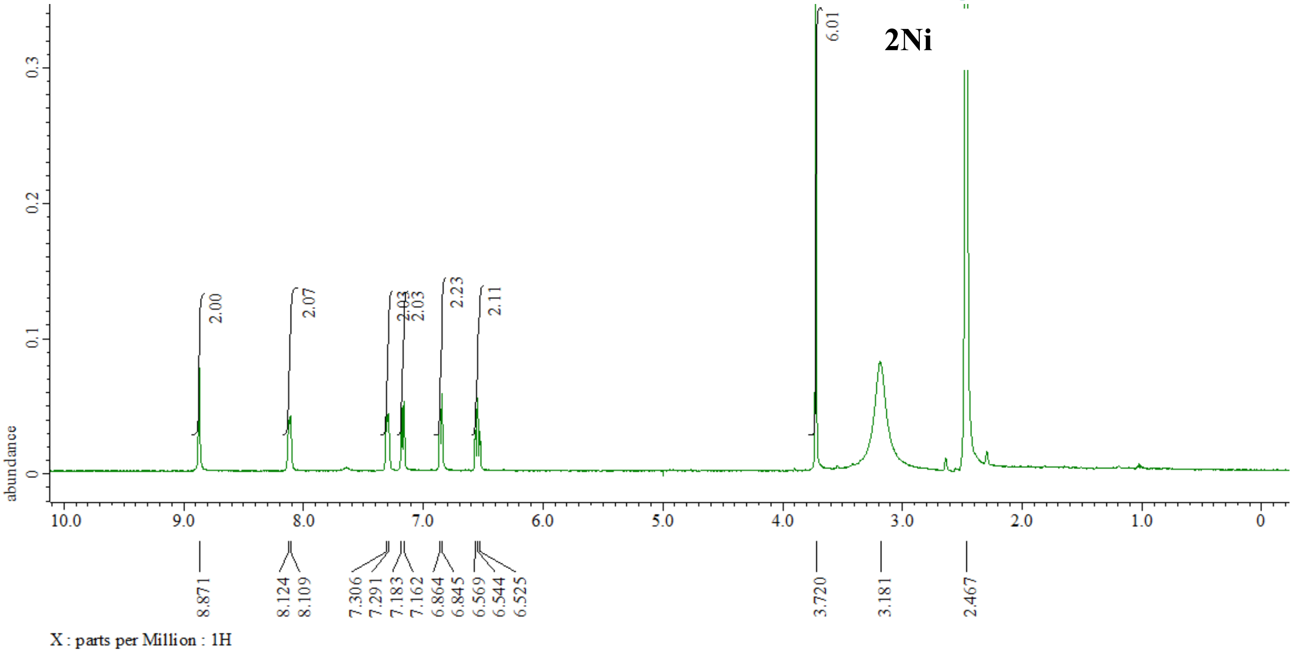

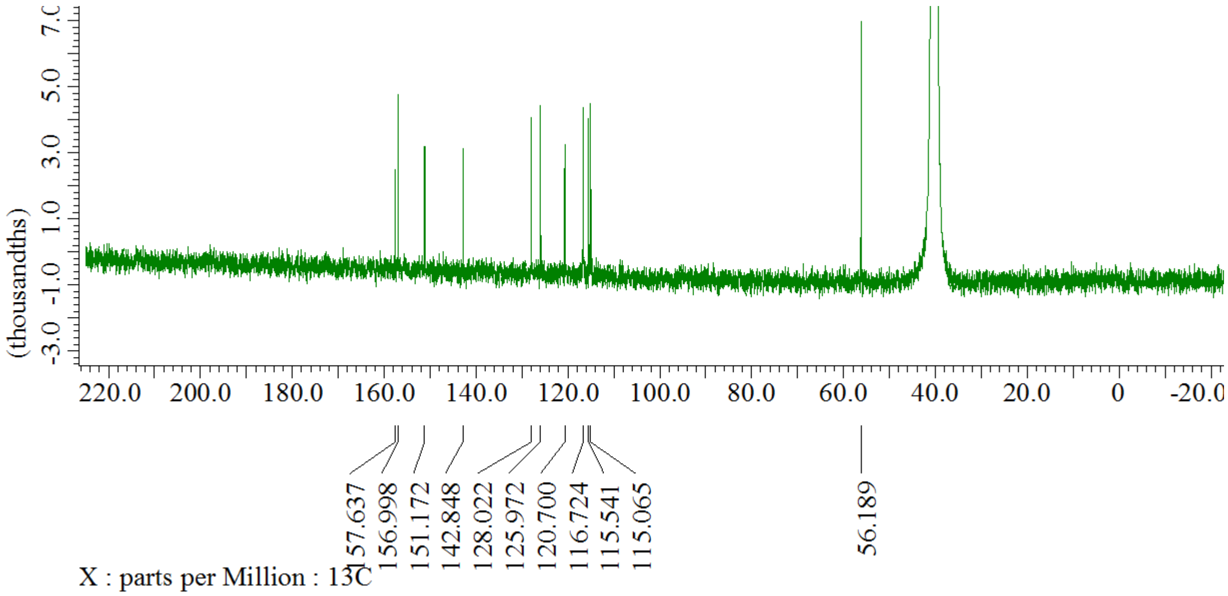

Figure S6. ^{1}H NMR (400 MHz, DMSO-d_6) and ^{13}C-NMR (100 MHz, DMSO-d_6) spectra of compound **2Ni**. ^{1}H NMR δH (400 MHz, DMSO-d_6), 8.87 (2H, s, CH), 8.12-8.10 (2 H, m, CH), 7.30-7.29 (2 H, m, CH), 7.17 (2 H, d, CH, J = 8Hz), 6.85 (2 H, d, CH, J = 8Hz), 6.54 (2 H, t, CH, J = 8 Hz), 3.72 (6H, s, OCH3) . ^{13}C NMR δC (100 MHz, DMSO-d_6) 157.6, 156.9, 151.2, 142.8, 128.0, 125.9, 120.7, 116.7, 115.5, 115.1, 56.2).

Compound 2Pd

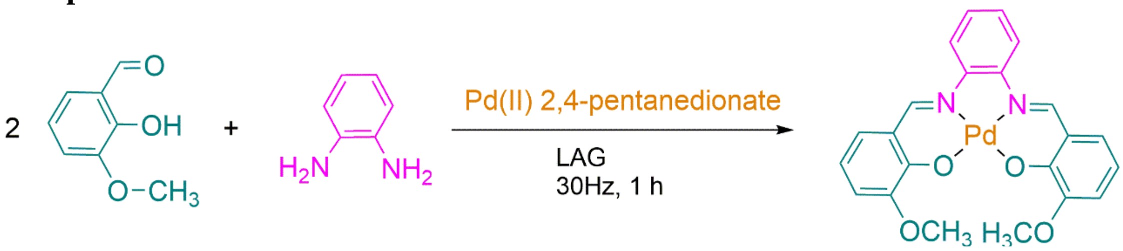

2Pd

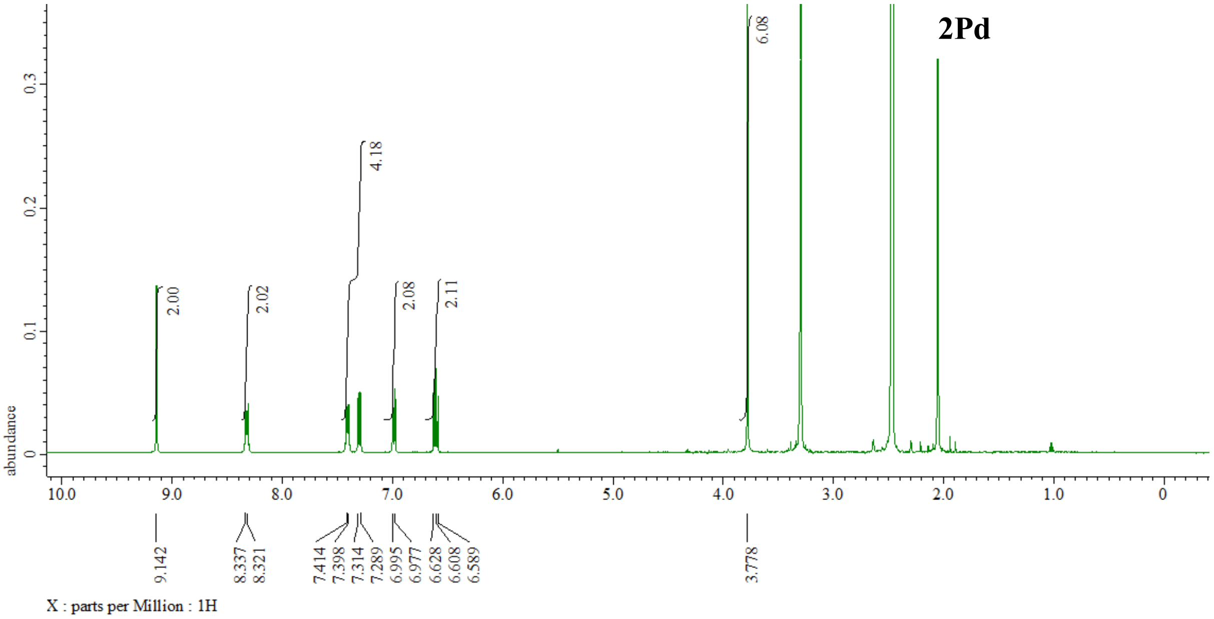

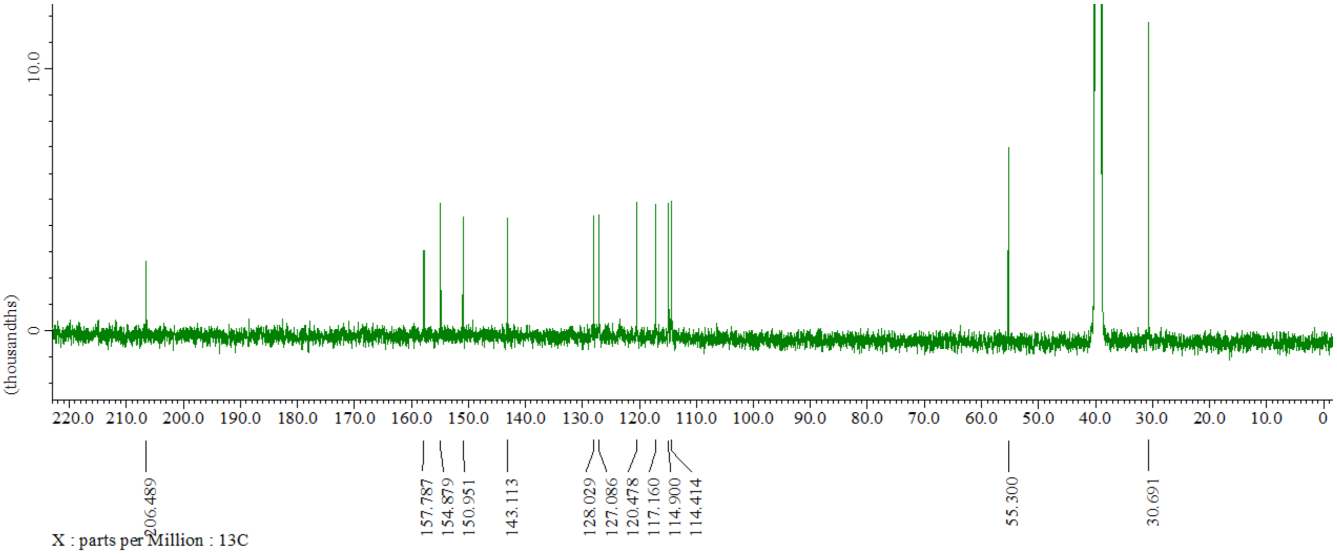

Figure S7. ^{1}H NMR (400 MHz, DMSO-d_6) and ^{13}C-NMR (100 MHz, DMSO-d_6) spectra of compound **2Pd**. Peaks at 206 and 31 ppm is acetone (used to wash the NMR tubes). ^{1}H NMR δH (400 MHz, DMSO-

d6), 9.14 (2H, s, CH), 8.33-8.32 (2 H, m, CH), 7.41-7.39 (2 H, m, CH), 7.29 (2 H, d, CH, J = 8Hz), 6.98 (2 H, d, CH, J = 8Hz), 6.60 (2 H, t, CH, J = 8 Hz), 3.78 (6H, s, OCH3) . ^{13}C NMR δC (100 MHz, DMSO-*d6*) 157.8, 154.9, 150.9, 143.1, 128.0, 127.1, 120.5, 117.2, 114.9, 114.4, 55.3).

Compound 3Zn

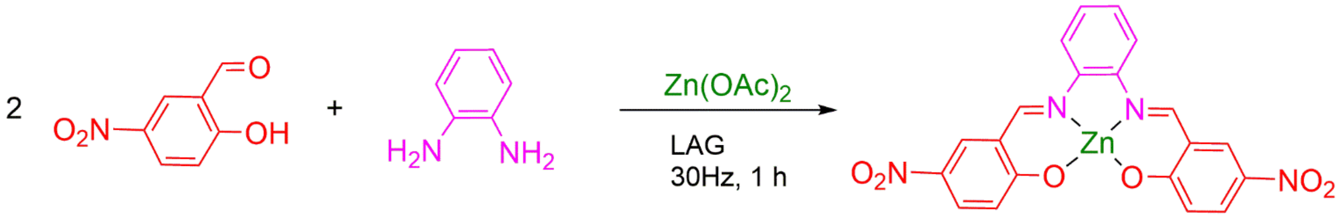

3Zn

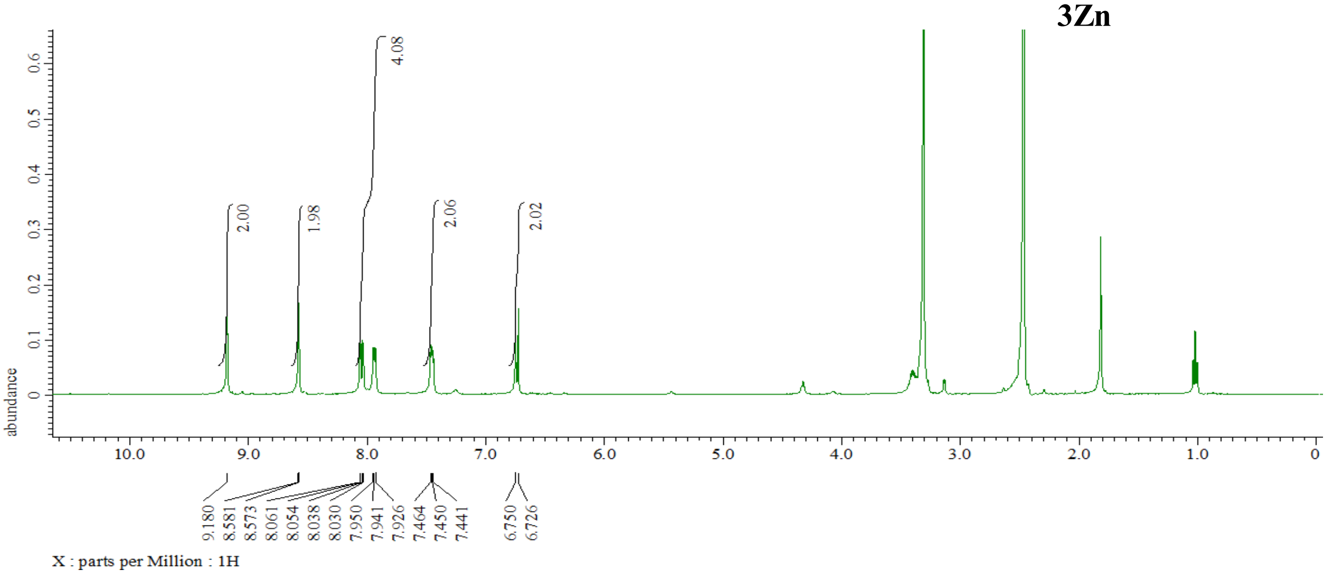

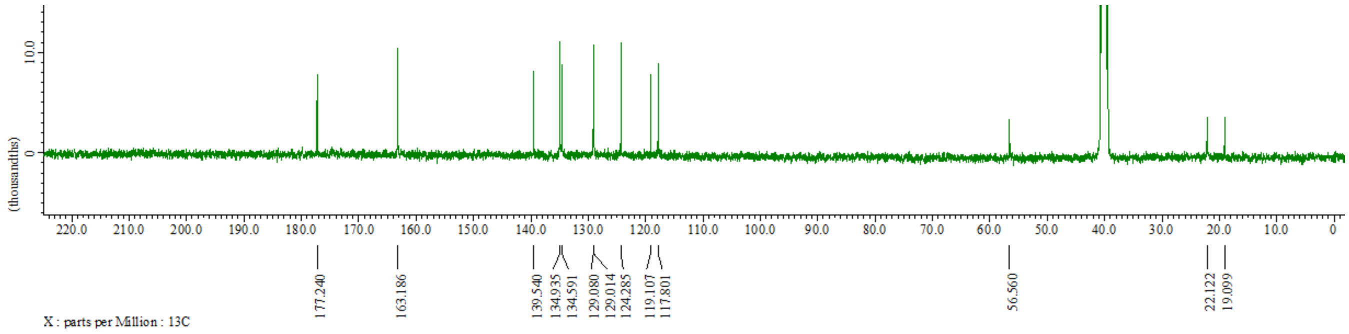

Figure S8. ^{1}H NMR (400 MHz, DMSO-*d6*) and ^{13}C-NMR (100 MHz, DMSO-*d6*) spectra of compound **3Zn**. Peaks at 206 and 31 ppm is acetone (used to wash the NMR tubes). ^{1}H NMR δH (400 MHz, DMSO-*d6*), 9.18 (2H, s, CH), 8.57 (2 H, d, CH, J = 3Hz), 8.05 (2 H, dd, CH, J1 = 8Hz, J2 = 3Hz), 7.94 (2 H, m, CH), 7.45 (2 H, m, CH), 6.74 (2 H, d, CH, J = 8 Hz). ^{13}C NMR δC (100 MHz, DMSO-*d6*) 177.2, 163.2, 139.5, 134.9, 134.6, 129.1, 129.0, 124.3, 119.1, 117.8).

NMR spectra of this compound is in agreement with that reported in the literature. [5]

SSNMR compound 2 and complexes 2Zn, 2Ni.

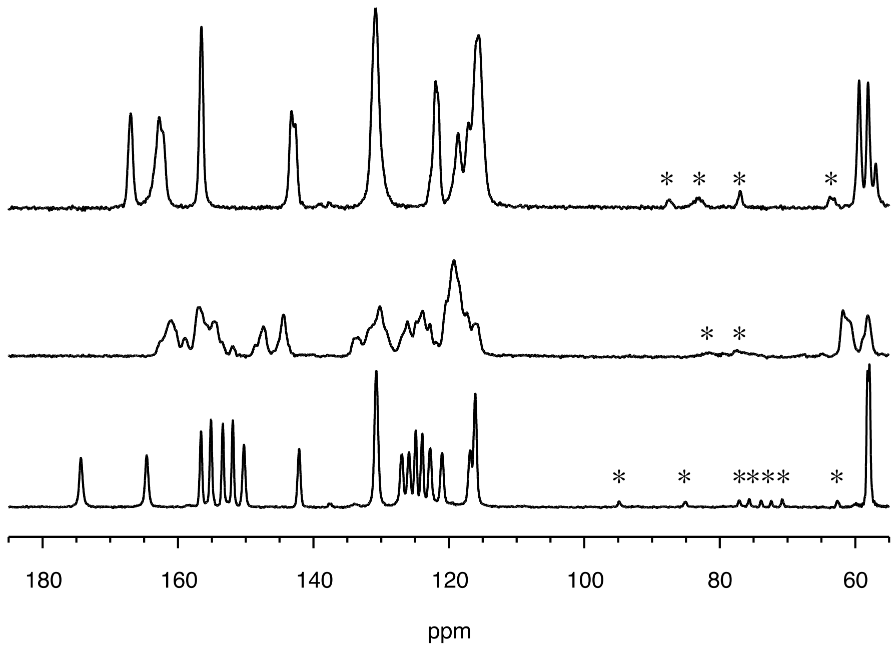

Figure S10. SSNMR spectra recorded at 11.7 T and room temperature of mechansynthesized salophen and metal-salophens (From top to bottom: **2Zn**, **2Ni**, and compound **2**). Spinning sidebands are indicated by asterisks.

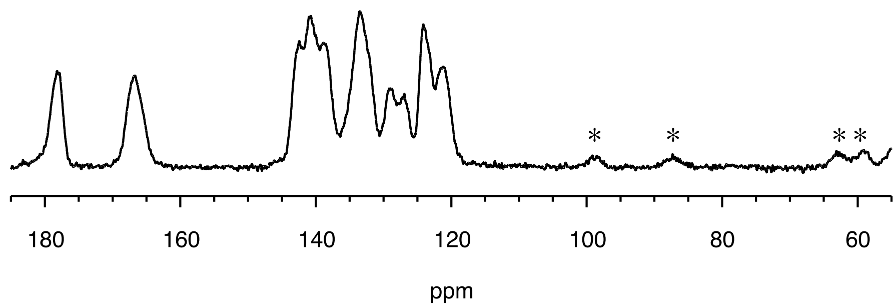

Figure S11. SSNMR spectra recorded at 11.7 T and room temperature of compound **3Zn**. Spinning sidebands are indicated by asterisks.

Table S1. Crystallographic data for reported compounds.

	**1Pd·(DMSO)$_2$**	**2Ni·DMSO**	**2Pd·DMSO**	**3Zn·(DMSO)$_2$**
**Chemical formula**	$C_{20}H_{14}N_2O_2Pd$· 2(C_2H_6OS)	$C_{22}H_{18}N_2NiO_4$· C_2H_6OS	$C_{22}H_{18}N_2O_4Pd$· C_2H_6OS	$C_{20}H_{12}N_4O_6Zn$· 2(C_2H_6OS)
M_r	576.99	511.22	558.91	625.96
**Crystal system, space group**	Monoclinic, $P2_1/n$	Monoclinic, $P2_1/c$	Monoclinic, $P2_1/c$	Monoclinic, $P2_1/c$
**Temperature (K)**	100	100	100	295
***a*, *b*, *c* (Å)**	14.9259 (15), 5.7838 (4), 27.112 (3)	12.1801 (4), 16.3689(2), 11.9448 (2)	12.2585 (3), 16.5750(3), 11.8022 (2)	26.8146 (8), 11.8980 (3), 8.3797 (3)
**β (°)**	100.176 (12)	106.250 (3)	105.750 (2)	91.447 (3)
V (Å^3)	2303.7 (4)	2286.35 (10)	2308.01 (8)	2672.60 (14)
Z	4	4	4	4
**Radiation type**	Mo $K\alpha$	Mo $K\alpha$	Mo $K\alpha$	Cu $K\alpha$
**μ (mm^{-1})**	1.02	0.98	0.93	3.22
**Crystal size (mm)**	0.49 × 0.06 × 0.04	0.28 × 0.18 × 0.06	0.26 × 0.18 × 0.16	0.46 × 0.21 × 0.02
T_{min}, T_{max}	0.907, 0.961	0.817, 0.947	0.838, 0.882	0.501, 0.951
R_{int}	0.032	0.026	0.026	0.034
**(sin θ/λ)$_{max}$ (Å^{-1})**	0.714	0.763	0.763	0.597
R*, *wR*, *S	0.033, 0.068, 1.03	0.036, 0.088, 1.0 2	0.031, 0.073, 1.0 4	0.062, 0.179, 1.12
**No. of reflections**	7029	7603	7717	4711
**No. of parameters**	302	302	302	434
**H-atom treatment**	H-atom parameters constrained	H-atom parameters constrained	H-atom parameters constrained	H-atom parameters constrained
**Δρ$_{max}$, Δρ$_{min}$ (e·Å^{-3})**	0.60, −0.60	0.50, −0.44	0.62, −0.54	0.67, −0.40
**CCDC deposition number**	1844978	1844979	1844980	1844981

Table S2. Bond distances of the compounds under study

Compound	O1…C6	C6…C1	C1…C7	C7…N	N…M	M…O1	M…O (DMSO)
**1**	1.352(3)	1.404(3)	1.445(3)	1.288(3)	/	/	/
	1.348(3)	1.404(3)	1.454(3)	1.278(3)	/	/	/
**2**	1.347(2)	1.397(2)	1.440(3)	1.281(2)	/	/	/
	1.353(2)	1.388(3)	1.455(3)	1.277(2)	/	/	/
**2Ni·DMSO (M = Ni)**	1.314(2)	1.410(2)	1.419(2)	1.308(2)	1.860(1)	1.857(1)	/
	1.305(2)	1.415(2)	1.417(2)	1.311(2)	1.859(1)	1.853(1)	/
**1Pd·(DMSO)$_2$ (M = Pd)**	1.312(3)	1.430(3)	1.420(3)	1.307(3)	1.963(2)	1.982(1)	/
	1.311(2)	1.425(3)	1.429(3)	1.303(3)	1.960(2)	1.990(2)	/
**2Pd·DMSO (M = Pd)**	1.306(2)	1.419(2)	1.425(2)	1.305(2)	1.959(2)	1.984(1)	/
	1.314(2)	1.419(2)	1.425(2)	1.304(2)	1.959(1)	1.995(1)	/
**3Zn·(DMSO)$_2$ (M = Zn)**	1.279(5)	1.449(6)	1.431(7)	1.278(6)	2.095(3)	1.976(3)	/
	1.284(6)	1.435(7)	1.446(6)	1.293(5)	2.077(4)	1.995(4)	2.150(9)

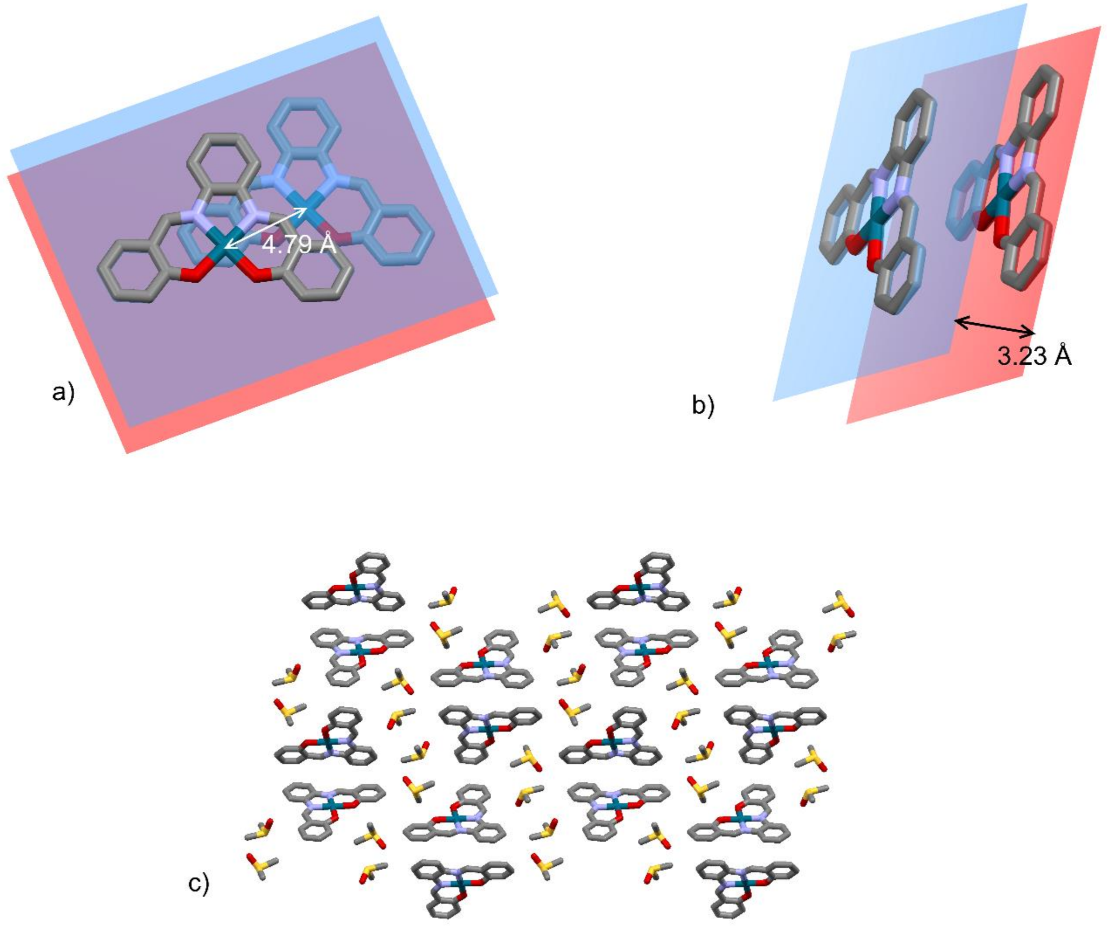

Figure S12. Head-to-head dimers in **1Pd·(DMSO)$_2$** with measured parallel displacement (a) as well as interplanar separation (b). View of crystal packing (c).

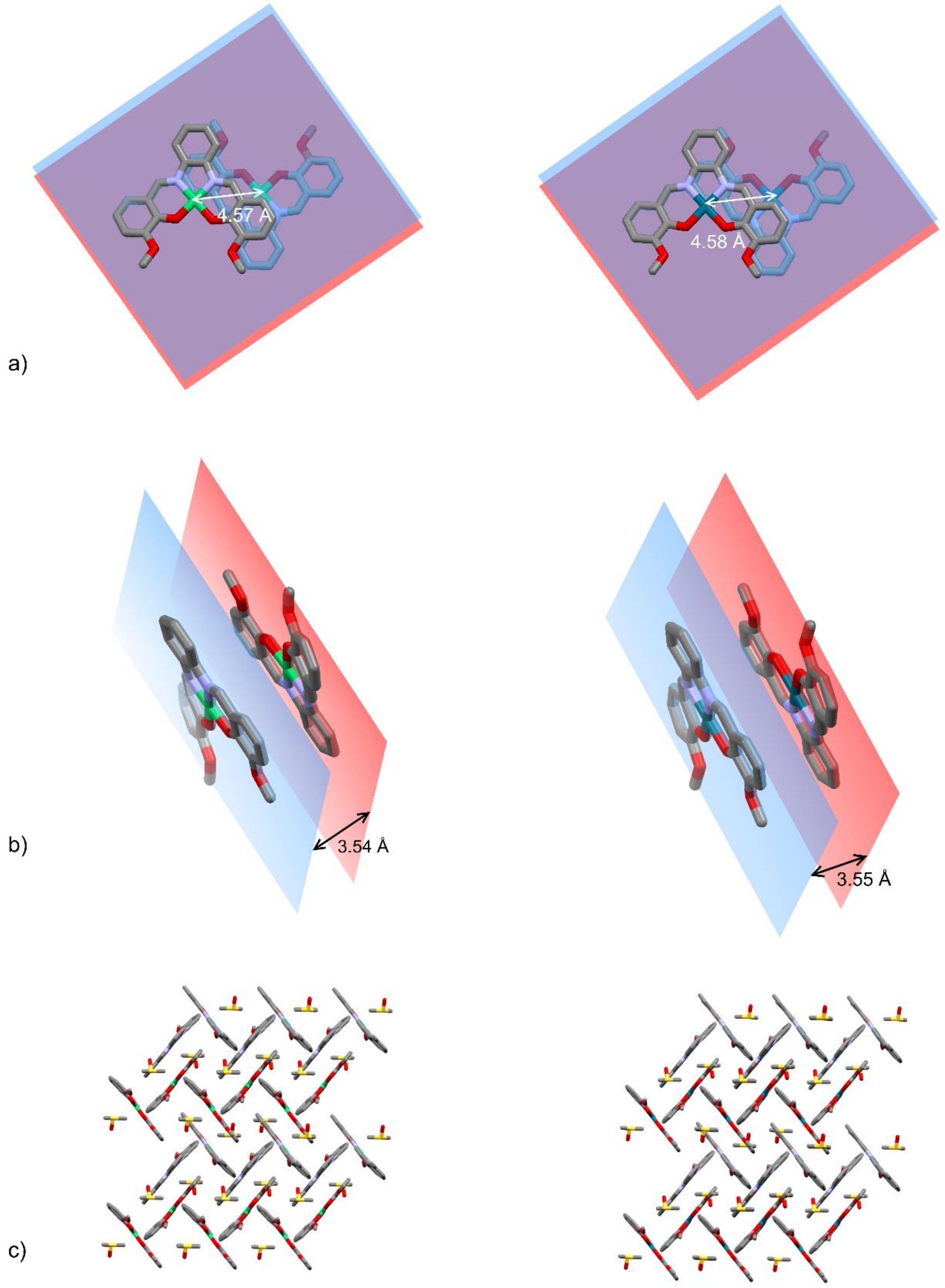

Figure S13. Head-to-tail dimers in **2Ni·DMSO** (left) and **2Pd·DMSO** (right) with measured parallel displacement (a) as well as interplanar separation (b). View of crystal packing (c).

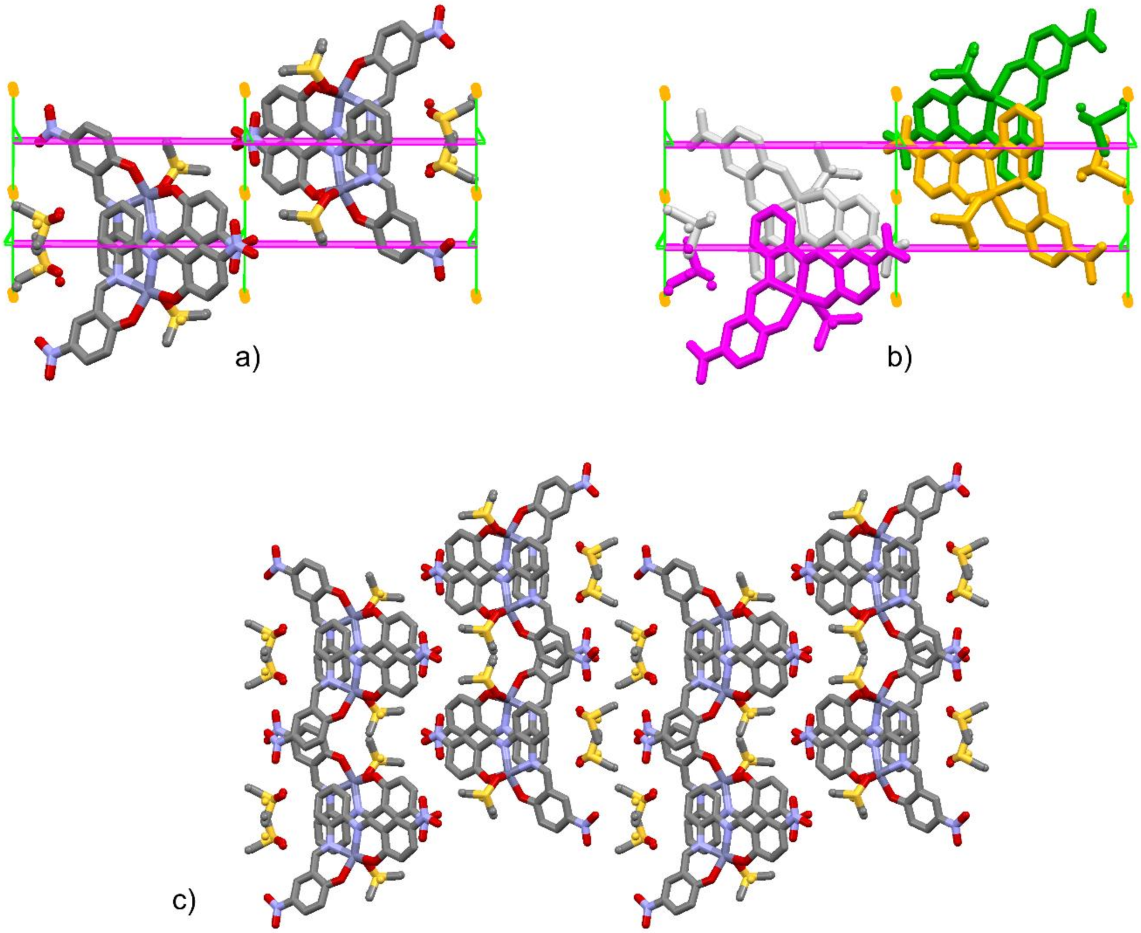

Figure S14. Unit cell of **3Zn·(DMSO)$_2$** with highlighted symmetry elements (a, purple = glide plane, green = 2-fold screw axes, yellow spots = inversion centers) and molecules colored in accordance to the symmetry operation (b). View of crystal packing (c).

Table S3. Selected geometries for the distorted square pyramid observed in **3Zn·(DMSO)$_2$** (M = Zn). The equatorial plane is calculated from the atomic coordinates of N1, N2, O1, O2.

Distances				Angles	
**d(N1…M)<br>d(N2…M)**	**d(O1…M)<br>d(O2…M)**	**d(O$_{(DMSO)}$…M)**	**M…(equatorial plane)**	**O$_{(DMSO)}$-M-N1<br>O$_{(DMSO)}$-M-N2**	**O$_{(DMSO)}$-M-O1<br>O$_{(DMSO)}$-M-O2**
2.095(3) Å	1.976(3) Å	2.150(9) Å	0.362(3) Å	93.5(3)°	101.0(3)°
2.077(4) Å	1.995(4) Å			105.1(3)°	101.2(3)°

References

[1] L. Jiang, Neng, X. Su-Yi, L. Sai-Sai, W. Zhong-Rui, W. Kelvin D.G., K. Xiao-Bing, *European Journal of Medicinal Chemistry*, 2014, **87**, 540.

[2] J. Lai, X. Ke, J. Tang, J. Zhang, *Chinese Chemical Letters*, 2015, **26**, 937.

[3] K. J. Miller, J. H. Baag,; M. M. Abu-Omar, *Inorganic Chemistry*, 1999, **38**, 4510.

[4] W. Lo, W. Wong, W. Wong, J. Guo, K. Yeung, Y. Cheng, X. Yang, and R. A. Jones, *Inorganic Chemistry*, 45, 2006, **23**, 9315.

[5] I. Giannicchi, R. Brissos, D. Ramos, J. de Lapuente, J. C. Lima, A. Dalla Cort and L. Rodríguez, *Inorg. Chem.,* 2013, **52** , 9245.

[6] P. E. Reyes-Gutiérrez, T. Kapal, B. Klepetářová, D. Šaman, R. Pohl, Z. Zawada, E. Kužmová, M. Hájek, F. Teplý, *Sci. Rep.* 2016, **6**, 23499.

[7] W.-K. Lo, W.-K. Wong, W.-Y. Wong, J. Guo, K.-T. Yeung, Y.-K. Cheng, X. Yang, R. A. Jones, *Inorg. Chem*., 2006, **45**, 9315.
